# Gut microbiome dysbiosis and associated metabolome changes in tuberculosis patients resolve partially post-therapy

**DOI:** 10.1186/s12866-026-05462-1

**Published:** 2026-11-12

**Authors:** Sukanya Sahu, Sandeep R Kaushik, Shweta Chaudhary, Amit Kumar Mohapatra, Rukuwe-u Kupa, Wetesho Kapfo, Sourav Saha, Ranjit Das, Nikhil Bhalla, Manjeet Kaur, Yuvaranjani Sivaji, Bhabatosh Das, Anjan Das, Vinotsole Khamo, Ranjan Kumar Nanda

**Affiliations:** 1 Translational Health Group, International Centre for Genetic Engineering and Biotechnology, New Delhi, India; 2 Healthcare Laboratory and Research Centre, Naga Hospital Authority, Kohima, Nagaland India; 3 Department of Respiratory Medicine, Agartala Government Medical College, Agartala, India; 4 Microbial Research Centre, BRIC - Translational Health Science and Technology Institute, Faridabad, Haryana India

**Keywords:** Tuberculosis, Gut microbiota, Dysbiosis, Fecal metabolite, GC-TOF-MS

## Abstract

**Background:**

*Mycobacterium tuberculosis* (Mtb) infection in the host and subsequent treatment with anti-tuberculosis drugs impact lung pathophysiology and influence the lung and gut microbiome. Elucidating the role of the gut microbiome and its metabolites in the perturbed metabolic and immune functions observed in tuberculosis (TB) might help design corrective prebiotic and probiotic mixtures.

**Methodology:**

In this study, fecal samples (*n* = 107) of drug-naïve active TB patients (ATB) and controls (other respiratory disease: NTB and healthy) from the two clinical sites were analyzed for microbiota composition (16S rRNA sequencing), metabolites (GC-TOF-MS), and their correlation. In addition, a longitudinal follow-up study was conducted to understand the dynamics of the microbiota and metabolites during anti-TB treatment in a small cohort (*n* = 10).

**Results:**

Gut microbiota showed site-specificity; however, an overall reduction in alpha diversity was observed in disease states compared with healthy subjects. Underrepresentation of gut commensals (*Faecalibacterium*, *Ligilactobacillus* and *Romboustia*) and overrepresentation of *Thomasclavelia* and *Clostridioides* were observed among active TB patients. The longitudinally followed-up ATB subjects undergoing treatment showed a time-dependent increase in beta-diversity variance. Over time, the beta diversity of follow-up subjects was almost similar to that of healthy controls. Decrease in the abundance of *Streptococcus* occurred after anti-TB treatment, with higher levels of opportunistic pathogens, such as *Escherichia* and *Stenotrophomonas*. Similarly, specific fecal metabolites also returned to pretreatment levels in relative abundance at later time points.

**Conclusions:**

The gut microbiota of TB patients showed dysbiosis at the time of case presentation and partially resolved upon complete therapeutic interventions. Partial recovery of microbiota observed during treatment is also reflected in the fecal metabolome, indicating the possible use of microbiome and metabolites as adjunct therapeutics in TB.

**Supplementary Information:**

The online version contains supplementary material available at https://doi.org/10.1186/s12866-026-05462-1.

## Background

Tuberculosis (TB) caused by *Mycobacterium tuberculosis* (Mtb) is still a global health epidemic. In 2024, around 10.7 million new cases of TB were reported globally, with approximately 1.23 million deaths associated with it (WHO report, 2025) [1]. About one-third of the world’s population is latently infected with Mtb, and statistically, about 10% of them might develop active TB (ATB) during their lifetime [2]. The Mtb pathogenesis and disease prognosis depend on multiple host and environmental factors, including the host gut microbiome. Recent advancements in genomics have enabled us to determine numerous difficult-to-culture microbes in biological matrices, enhancing the comprehensive characterization of microbiomes and their role in health and disease.

The presence of Mtb in the lungs alters the lung microbiome composition in TB patients. Similarly, the gut microbiota plays a critical role in maintaining a healthy immune-metabolic system and is significantly altered in many systemic and respiratory disease conditions, such as TB [3–10]. The impact of anti-TB drugs on organ function is well known; however, their effects on gut microbiota composition and their function remain largely unexplored. As the gut microbiota plays a key role in maintaining a healthy immune system, its dysbiosis can compromise immunity. Therefore, restoring depleted beneficial microbes or supplementing their metabolic products in diseased conditions like TB might directly modulate the host immune response to fight TB, improve prognosis, and reduce the incidence of drug resistance [11–13]. Current knowledge of gut microbiota dysbiosis in TB is limited to murine models and patients from specific settings, and there is limited information on the effects of long-term anti-TB therapy. However, antibiotics and other drugs are known to alter gut microbial composition, either directly or indirectly [14, 15]. Thus, understanding their impact on gut microbial composition is essential for elucidating host–microbiome interactions, optimizing therapeutic responses, and improving clinical outcomes.

In the current study, fecal microbiome and metabolome profiling of drug-naïve pulmonary ATB patients were compared with healthy and non-tuberculosis (NTB) controls to identify biologically significant microbes and metabolic products. A longitudinal follow-up study of ATB patients who completed 6 months of treatment was also conducted to capture the dynamics during anti-TB treatment. The study identified a set of microbiota and metabolites that could be supplemented as an adjunct to anti-TB drugs to improve prognosis.

## Methodology

### Study approval

The study was performed in accordance with relevant guidelines and regulations and approved by the institute review boards of Naga Hospital Authority, Kohima (NHAK) (NHAK/HLRC-008/2012 dated 17th May 2017), Agartala Government Medical College, Agartala (AGMC) (protocolF.4[6–9]/AGMC/Academic/IEC Committee/2015/8965, dated 25 April 2018), and International Centre for Genetic Engineering and Biotechnology, New Delhi (ICGEB/IEC/2017/07). Written informed consent was obtained from all participants before the collection of biological samples. This study included both male and female subjects. All experiments were performed in accordance with the guidelines of the partnering institutes.

### DNA extraction and 16S rRNA gene amplicon sequencing

Genomic DNA was extracted from fecal samples using previously reported methods with minor modifications and stored at -20 °C until further analysis [16]. The quality and integrity of the extracted genomic DNA were assessed by agarose gel electrophoresis, and then sent to a commercial laboratory (Macrogen, South Korea) for library preparation and sequencing. Briefly, the hypervariable region V3-V4 of the bacterial 16S rRNA gene was amplified with the forward (Bakt_341F: CCTACGGGNGGCWGCAG) and the reverse primer (Bakt_805R: GACTACHVGGGTATCTAATCC). The 300 bp PE V3-V4 amplicon libraries were sequenced using Illumina MiSeq. Detailed protocols for DNA extraction, library preparation, and sequencing are provided in the Supplemental Experimental Procedures.

### Sequence data processing

The Quantitative Insights into Microbial Ecology (QIIME2 v.2024.5) tool was used to analyse the 16S rRNA sequencing data [17]. Non-singleton amplicon sequence variants (ASVs) were generated after quality filtering, denoising, merging, and chimaera removal using the DADA2 plugin. DADA2 summary statistics table of metagenomics data is presented in Supplementary tables S3, S4 and S5. Taxonomy was annotated with the NCBI-RefSeq database and classified at the level of phylum, class, order, family and genus. For the non-parametric test, Linear Discriminant Analysis with Effect Size (LEfSe) was performed on the relatively normalized ASV Table [18]. Functional profiles were predicted from 16S rRNA datasets using Phylogenetic Investigation of Communities by Reconstruction of Unobserved States (PICRUSt2) software and the Kyoto Encyclopedia of Genes and Genomes (KEGG) database [19]. Metabolic pathway analysis used PICRUSt2 to predict perturbed KEGG pathways in samples. The predicted pathways were grouped into parent classes based on the KEGG BRITE hierarchy and visualized using R (v 4.5.3).

### Fecal metabolite extraction and derivatization for GC-MS analysis

Lyophilized fecal samples (10.0 ± 0.3 mg; mean ± standard deviation, *n* = 103) were extracted with chilled methanol (1.0 ± 0.3 ml, − 20 °C) along with lysine D4 (5 µl of 10 mg/ml) as a spike in standard. The sample mixture was vortexed (1 min) and subjected to ultrasonication at 20% amplitude (30 s on/off cycle, total time 3 min), using QSonica Q500 sonicators (USA). The samples were vortexed and centrifuged at 16,000 g for 15 min at 4 °C. A fraction of the supernatant (300 µl) was transferred to a microcentrifuge tube (MCT) and vacuum dried at 40 °C in a SpeedVac vacuum concentrator (Labconco, USA). Aliquots of extracted metabolites from all the fecal samples (*n* = 103) were pooled to prepare a quality control (QC). Derivatization of the fecal metabolites was carried out using previously reported methods, as described in detail in the Supplemental Experimental Procedures [20].

### GC-MS data acquisition, processing, and analysis

Using an automated multipurpose sample introduction system (MPS, Gerstel, Germany), the derivatized fecal metabolites (1 µl) were loaded into the column attached to a GC-time of flight (TOF)-MS instrument (Pegasus 4D; Leco, USA) in splitless injection mode. Helium was used as the carrier gas at a constant flow rate of 0.5 ml/min for each run. Metabolite separation was carried out in an HP-5MS column (30 m × 0.25 mm × 0.25 μm; Agilent Technologies) with a temperature gradient from 60 °C to 300 °C at a ramp of 10 °C/min (60 to 220 °C) and 5 °C/min (220 to 300 °C). Hold times of 1 min and 5 min were kept at the start and end of the run, respectively. The injection port temperature was set at 250 °C throughout the analysis. Mass spectrometric data acquisition was performed at -70 eV, and a mass range of 50–550 m/z was scanned at 50 spectra/sec. A 575 s solvent delay was used, and the source temperature was set at 220 °C. All GC-MS parameters were controlled using ChromaTOF software (V.4.50.8.0; Leco, USA), and data acquisition was completed. Raw GC-MS data files (*n* = 112) of all the QC (*n* = 9) and study samples (*n* = 103) were pre-processed and aligned using the “Statistical Compare” feature of ChromaTOF. The minimum peak width was set to 1.3 s for peak picking, and the signal-to-noise ratio (S/N) threshold was set to 75. For tentative molecular feature identification, mainlib, nist_ri, nist_msms2, nist_msms, and replib libraries from NIST were used. The maximum retention time difference was set to 0 s, and the minimum spectral similarity was set to 600 for mass spectral match. Aligned peak information was exported to a “.csv” format, and molecules present in more than 50% of samples in at least one study group were included in the analysis. Manual data curation was done to align peaks and remove unsilylated molecules and silanes from the data matrix.

MetaboAnalyst 6.0 was used for univariate and multivariate statistical analyses, with the identified molecular features as variables and their abundance. Missing values of variables were imputed with one-fifth of the minimum positive values. Normalization by reference feature, i.e., lysine D4 with auto-scaling, was done to obtain a near-normal distribution. A partial least squares discriminant analysis (PLS-DA) model was built using metadata containing all the study samples and all qualifying molecular features. Analytes with VIP scores > 1.0 in the PLS-DA were selected as important features for classifying the study groups. For important feature selection, a fold change (FC) of at least 2.0 (log_2_FC > ± 1.0), with a Student’s t-test with p-value < 0.05 parameters, was selected. Most of the identified essential features were validated using commercial standards, following similar pre-processing and GC-MS methods.

### Metabolomics—microbiome data integration

The microbiome and metabolome data of the study subjects were integrated using the mixOmics R package (version 6.34.0) [21]. Differential metabolites and microbial taxa (at the genus level) identified among the ATB, NTB, and healthy groups were subjected to integrative correlation analysis. The mixOmics regularized canonical correlation analysis (rCCA) function generated clustered heatmaps. Euclidean distance was used to cluster the microbiome data in the rows and the metabolome data in the columns.

### Statistical analysis

Alpha diversity was characterised using the Shannon and Faith PD indices, and the Kruskal-Wallis (KW) H test was used to assess group-specific differences. Beta diversity was described using unweighted and weighted UniFrac distance matrices, and Permutational Analysis of Variance (PERMANOVA) was used to validate the differences between study groups. LefSe uses the KW sum-rank test, the pairwise Wilcoxon rank-sum test, and Linear discriminant analysis (LDA) to identify differentially abundant taxa. Differential abundance analysis of predicted KEGG pathways among the study groups was performed in R (version 4.5.3) using the Kruskal–Wallis (KW) H-test, followed by Dunn’s post hoc multiple-comparison test without multiple-testing correction.

## Results

### Study design, subject recruitment, and Socio-demographic details

**Fig. 1:**
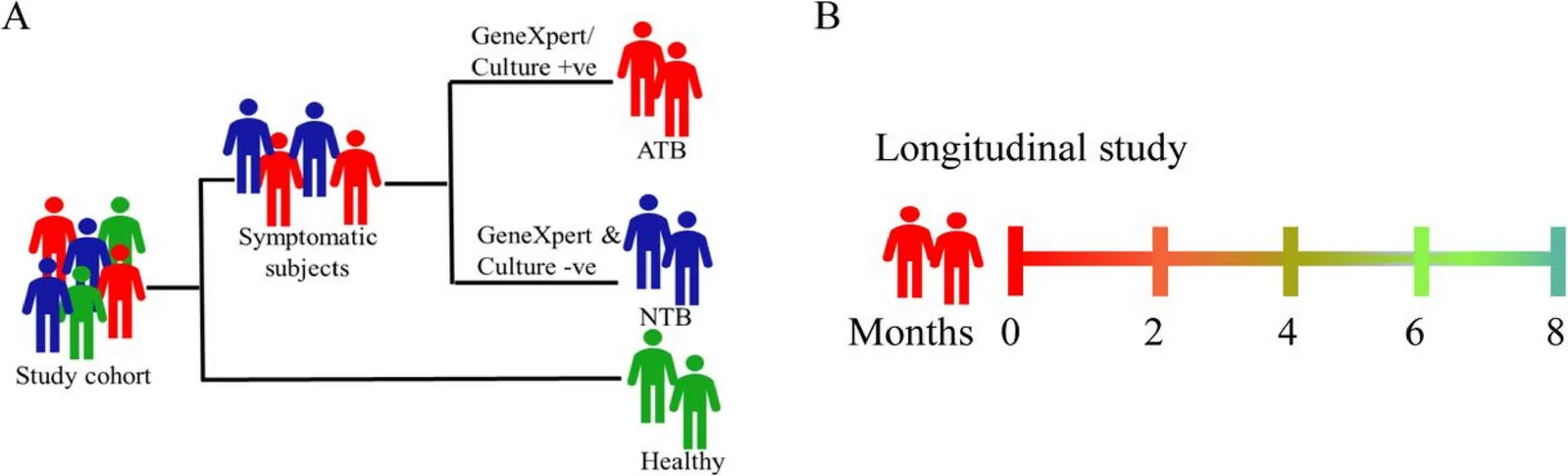
Strategy used for study subject recruitment and classification of (**A**) case-control and (**B**) longitudinally followed-up studies

Study subjects were recruited from Nagaland and Tripura, following protocols approved by the Nagaland Hospital Authority, Kohima (NHAK; site-I); Agartala Government Medical College, Agartala (AGMC; site-II); and the International Centre for Genetic Engineering and Biotechnology, New Delhi. Subjects reporting symptoms for 2 weeks of cough, fever, weight loss, and night sweats to the outpatient departments of NHAK and AGMC, as well as healthy subjects from the family members of these patients, were enrolled. After receiving signed informed consent forms, the subjects were enrolled in this study. Sputum samples (days 1 and 2) and fecal samples were collected on the day of reporting. Sputum samples were subjected to Ziehl-Neelsen acid-fast bacilli (AFB) microscopy, culture, and GeneXpert (Cepheid, USA) analyses following WHO guidelines at a National Accreditation Board for Testing and Calibration Laboratories (NABL). Subjects with positive microscopy results and culture or GeneXpert test results were grouped as active tuberculosis patients (ATB), and those with all negative test results were grouped as non-tuberculosis patients (NTB). An overview of volunteer recruitment is presented schematically in Fig. 1A. These NTB subjects were clinically diagnosed with other pulmonary disease conditions like asthma, chronic obstructive pulmonary disease (COPD), lung cancer, pneumonia, or suffering from more than one complication. The ATB patients received a 6-month anti-tuberculosis treatment regimen consisting of 2 months of intensive phase (Isoniazid, Rifampin, Pyrazinamide, Ethambutol; HRZE), followed by 4 months of continuation phase, as per the Revised National TB Control Programme (RNTCP) guidelines. A subset of ATB patients from site II was followed up longitudinally at 2, 4, 6, and 8 months after treatment initiation, and fecal samples were collected from these subjects at respective time-points (Fig. 1B). Fecal samples were stored at -80 ℃ within 4 h of collection until further processing. The sample size for each patient group in the study population was not calculated, as the study was primarily intended to generate preliminary data for future adequately powered investigations. Fecal samples from all three groups (ATB, NTB and healthy) were used for metagenomics and metabolomics. A total of 107 fecal samples were used for metagenomics (site-I, *n* = 45; site-II, *n* = 31; site-I follow-up, *n* = 31), and a subset of 94 samples used for metabolomics (site-I, *n* = 40; site-II, *n* = 30; site-I follow-up, *n* = 24). Demographic details of the study subjects are summarized in Supplementary Tables S1 and S2. Further, the site-I ATB subjects were relatively younger than healthy controls, whereas all the AGMC subjects had a similar age distribution (Supplementary Figures S1A, S1B).

### Microbial dysbiosis in ATB, NTB, and healthy control groups

**Fig. 2:**
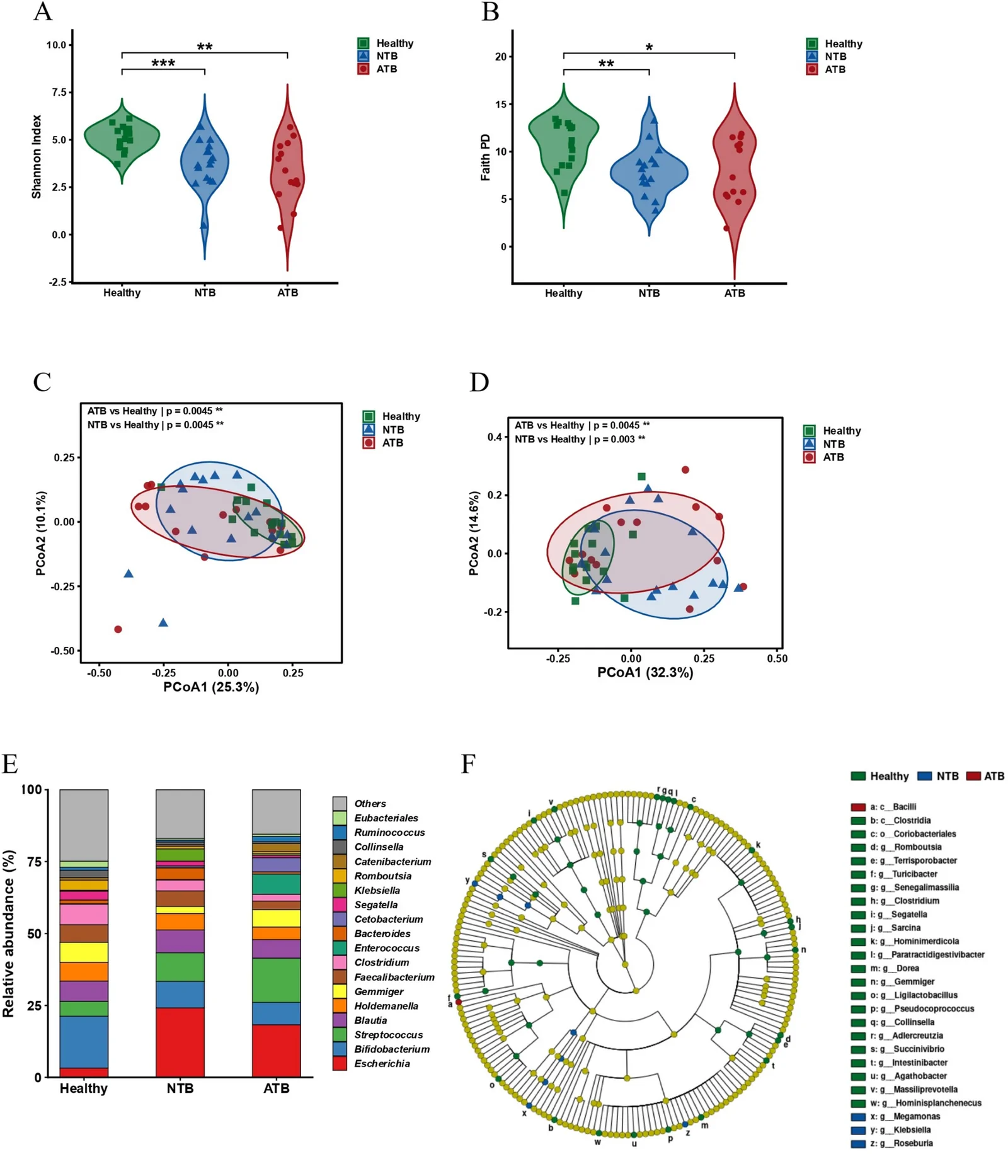
Gut microbial diversity analysis and mean relative abundance profiles among Healthy, NTB, and ATB groups from Site I. Alpha diversity comparisons based on (**A**) Shannon and (**B**) Faith PD indices. Principal coordinate analysis (PCoA) of (**C**) unweighted and (**D**) weighted UniFrac distances. *: *p* < 0.05; **: *p* < 0.01; ***: *p* < 0.001. (**E**) Stacked bar plots representing the mean relative abundance (%) of the most abundant microbial taxa collapsed to the genus level. Taxa with mean abundance < 1% and unclassified taxa were grouped as “Others”. (**F**) Cladogram representation of LEfSe analysis based on effect size (LDA score [log_10_] threshold of 2. Differences among study groups were assessed using the Kruskal-Wallis test (α = 0.05) and the Wilcoxon test (α = 0.05), with less stringent criteria for multi-class analysis

**Fig. 3:**
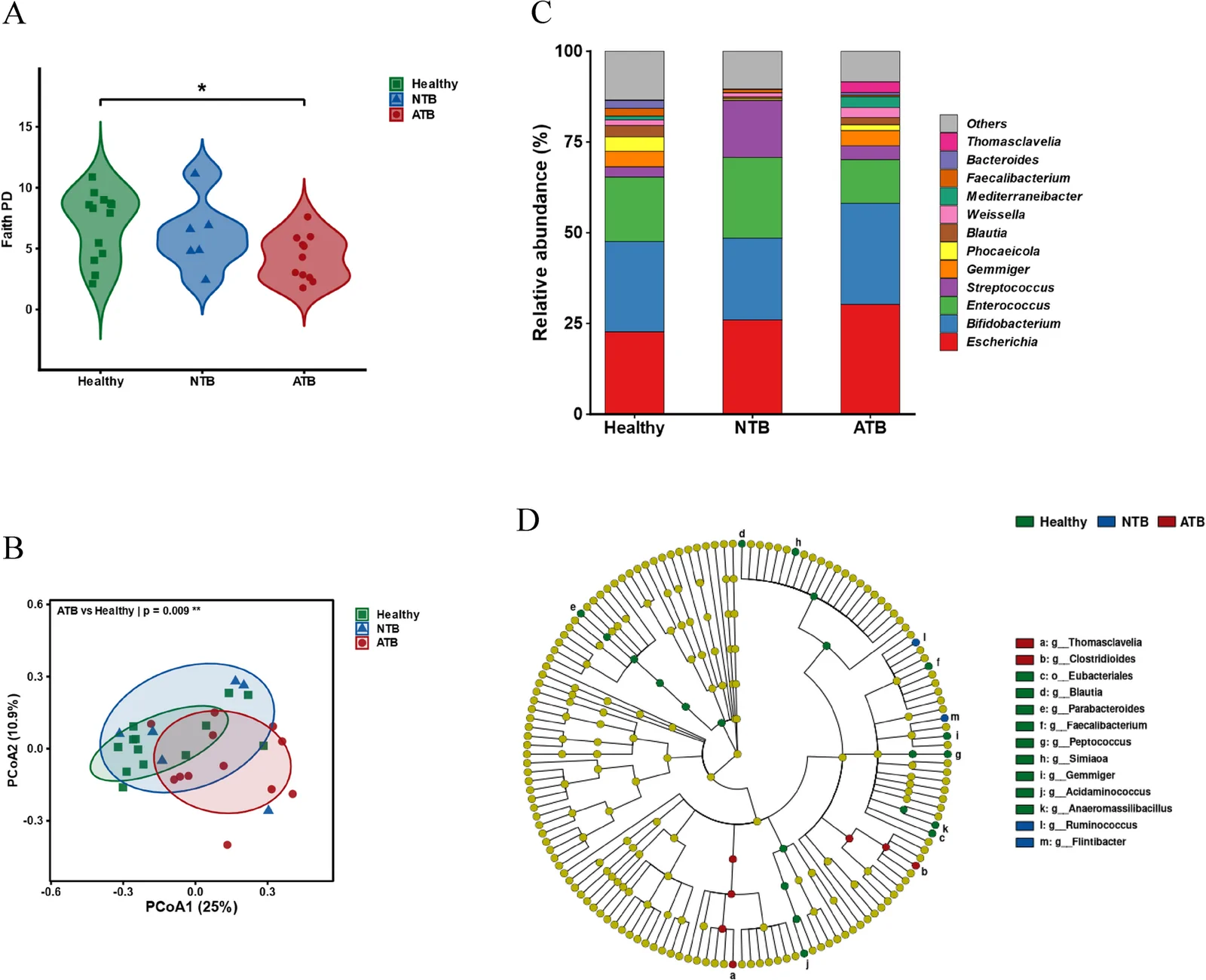
Gut microbial diversity analysis and mean relative abundance profiles among Healthy, NTB, and ATB groups from Site II. (**A**) Alpha diversity comparisons based on Faith PD index. (**B**) Principal coordinate analysis (PCoA) of unweighted UniFrac distance. *: *p* < 0.05; **: *p* < 0.01; ***: *p* < 0.001. (**C**) Stacked bar plots representing the mean relative abundance (%) of the most abundant microbial taxa collapsed to the genus level. Taxa with mean abundance < 1% and unclassified taxa were grouped as “Others”. (**D**) Cladogram representation of LEfSe analysis based on effect size (LDA score [log_10_] threshold of 2). Differences among study groups were assessed using the Kruskal-Wallis test (α = 0.05) and the Wilcoxon test (α = 0.05), with less stringent criteria for multi-class analysis

In total, 13,804,505 paired-end sequencing reads obtained from 107 fecal samples (an average of 1,27,894/sample) were processed. Statistically significant reductions in alpha diversity were observed in ATB and NTB patients compared to healthy subjects in NHAK (Site-I, Fig. 2A and B). In site-II, a significantly lower Faith PD index was observed in ATB compared to the healthy controls (Fig. 3A). Unweighted and weighted UniFrac analyses of site-I showed somewhat overlapped clustering of ATB and NTB from healthy subjects, which was statistically significant (unweighted; ATB/healthy: *p* = 0.0045, NTB/healthy: *p* = 0.0045; weighted; ATB/healthy *p* = 0.0045, NTB/healthy *p* = 0.003) (Fig. 2C and D). In site-II, the unweighted UniFrac analysis revealed a significant (*p* = 0.009) difference in alpha diversity between ATB and healthy subjects, whereas it was comparable between ATB and NTB groups (Fig. 3B).

A stacked taxa bar plot depicting the relative abundance profiles across experimental groups and LEfSe analysis of study subjects from site-I revealed that *Bacilli* was higher at the class level in ATB subjects, whereas an increased abundance of *Klebsiella* and *Megamonas* was observed in NTB subjects. *Escherichia’s* abundance is higher in both ATB and NTB subjects. A lower abundance of *Bifidobacterium*,* Romboustia*,* Segatella*,* Intestinibacter*,* Ligilactobacillus*,* Senegalimassila*,* Paratractidigestivibacter*,* Dorea*,* Terrisporobacter*, and *Collinsella* was observed in ATB and NTB subjects compared to healthy controls (Fig. 2E and F; Supplementary Figures S2A, S3, S4, S5). In site-II, *Thomasclavelia* and *Clostridioides* showed higher abundance in ATB subjects, whereas *Faecalibacterium and Parabacteroides* were reduced in ATB and NTB subjects compared to healthy (Fig. 3C and D; Supplementary Figures S2B, S6, S7).

### The fecal metabolome of ATB patients showed significant differences from controls

**Fig. 4:**
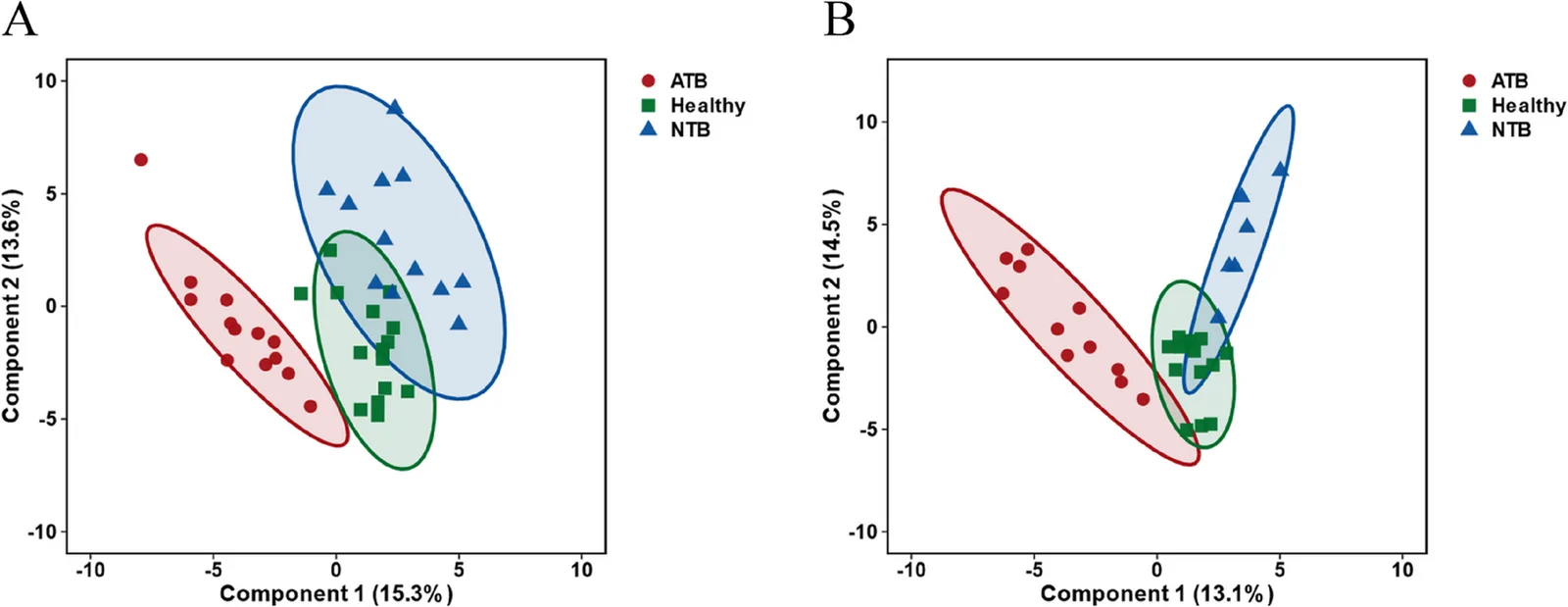
Fecal metabolites of active tuberculosis patients (ATB) showed significant variations from control (nontuberculosis: NTB and healthy) subjects. PLS-DA score plot showing group-specific clusters of fecal metabolites between ATB, Healthy and NTB Subjects of **A**) site-I and **B**) site-II. Coloured circles represent 95% confidence intervals. Coloured dots represent individual samples

Global fecal metabolome analysis of ATB, healthy, and NTB groups showed separate clusters between study groups from both study sites (Fig. 4A and B). Set of differentially (FC ≥ ± 2.0, *p* ≤ 0.05) deregulated metabolites between the study groups in NHAK: ATB/healthy (low/high:15/21), ATB/NTB (low/high:13/10), NTB/healthy (low/high:7/16); and in AGMC: ATB/healthy (low/high:6/28), ATB/NTB (low/high:8/12), NTB/healthy (low/high:4/25) were identified (Supplementary Figures S8A, S8C, S8E; S9A, S9C, S9E; Tables S6, S7, S8, S9, S10, S11). A set of top 25 molecular features qualifying a Variable Importance in Projection (VIP) score > 1.0 from the partial least square discriminant analysis (PLS-DA) was identified as important (Figure S8B, S8D, S8F; S9B, S9D, S9F). A significant decrease in SCFA (valeric acid) level was observed in the ATB group compared to controls, whereas organic acid (malonic acid) levels were significantly increased. N-acetyl putrescine level was significantly higher in the ATB group compared to the healthy group, whereas tyramine levels were lower in the ATB group when compared to the control group. Furthermore, a significant decrease in oleic acid levels was observed in ATB subjects compared with controls.

**Fig. 5:**
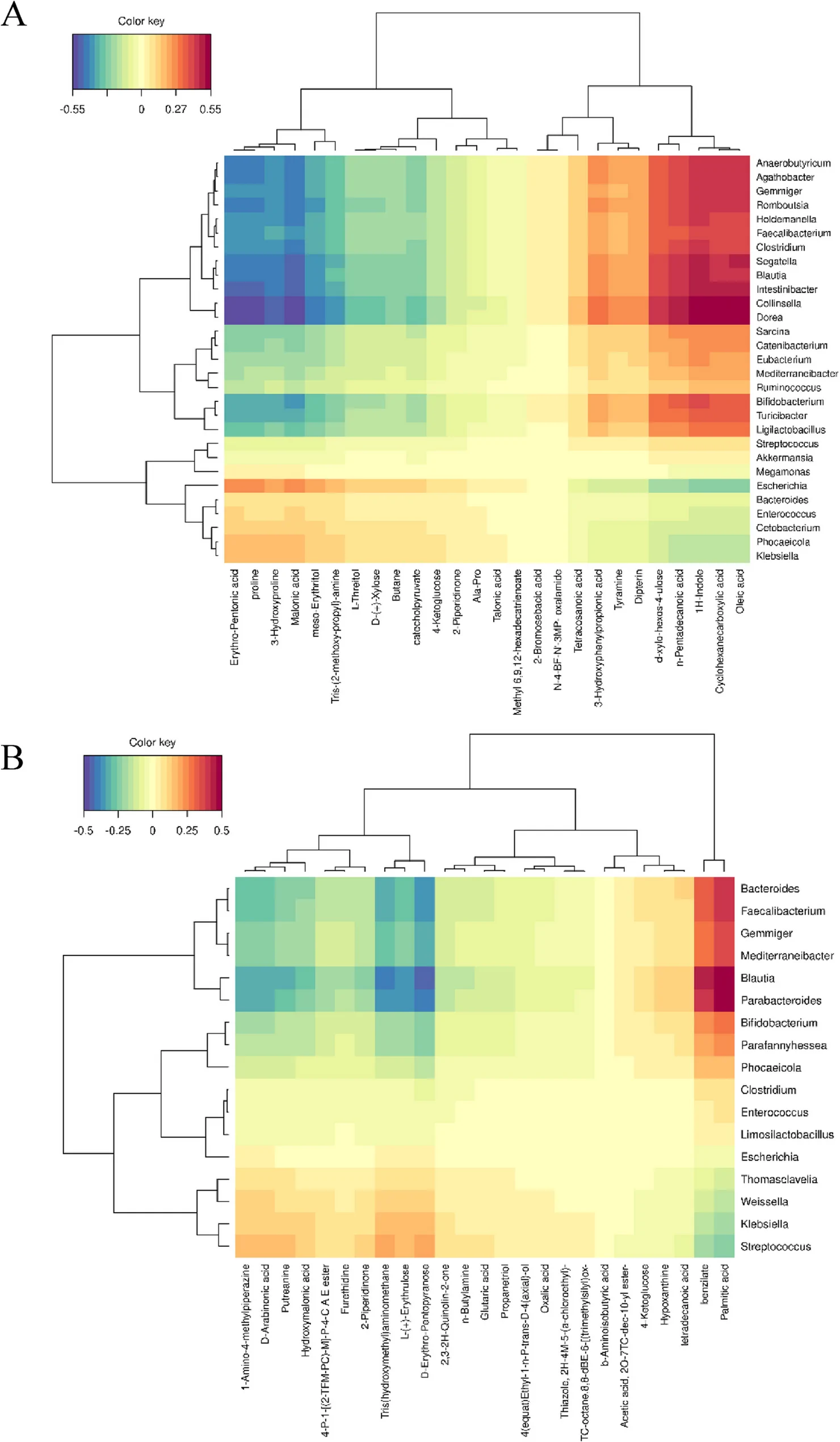
MixOmics analysis of study subjects from (**A**) site-I and (**B**) site-II. Clustered Image Map (CIM) depicting correlation between differential fecal metabolic patterns and bacterial genera in ATB, healthy, and NTB subjects. Regularized canonical correlation coefficients between the level of reliability and markedly different fecal metabolic patterns and the abundance of the differentially enriched bacteria were calculated. Blue: negative correlation; Red: positive correlation. N-4-BF-N’-3MP- Oxalamide: N-(4-Bromo-2-fluoro-phenyl)-N’-(3-morpholin-4-yl-propyl)-oxalamid; Thiazole, 2 H-4 M-5-(a-chloroethyl)-: Thiazole, 2- hydroxy-4-methyl-5-(a-chloroethyl)-; Acetic acid, 2O-7TC-dec-10-yl ester-: Acetic acid, 2-oxa-7-thia- tricyclo[4.3.1.0(3,8)]dec-10-ylester; 4-P-1-[(2-TFM-PC)-M]-P-4-C A E ester: 4-Phenyl-1-[(2-trifluoromethyl-phenylcarbamoyl)-methyl]-piperidine-4-carboxylic acid ethyl ester; TC-octane,8,8-dBE-6-[(trimethylsilyl)oxy]-: Tetracyclo[5.1.0.0(2,4)0.0(3,5)]octane, 8,8-dibromo-exo-6-[(trimethylsilyl)oxy]-

In site-I/NHAK, oleic acid and n-pentadecanoic acid exhibited a strong positive correlation with *Dorea*,* Collinsella*,* Intestinibacter* and *Segatella*, whereas proline, 3-hydroxyproline, and malonic acid showed strong negative correlations with these genera. Further, *Blautia*,* Clostridium*,* Faecalibacterium*,* Holdemanella*,* Gemmiger*,* Romboutsia*,* Agathobacter* and *Anaerobutyricum* displayed comparatively weaker but similar positive correlations with oleic acid and n-pentadecanoic acid, and negative correlations with proline, 3-hydroxyproline, and malonic acid (Fig. 5A). In site-II/AGMC, *Parabacteroides* and *Blautia* showed a strong positive correlation with palmitic acid and benzilate. In addition, *Mediterraneibacter*, Gemmiger, *Faecalibacterium*,* Bacteroides* and *Bifidobacterium* also displayed comparatively weaker but similar positive correlations with palmitic acid and benzilate. (Fig. 5B). Other significant correlations between microbes and metabolites as variables from both study sites are detailed in the clustered image maps (Fig. 5A and B).

### Longitudinal gut microbiota analysis revealed a trend toward a healthy-like configuration in gut metabolome at 8 months post-treatment

**Fig. 6:**
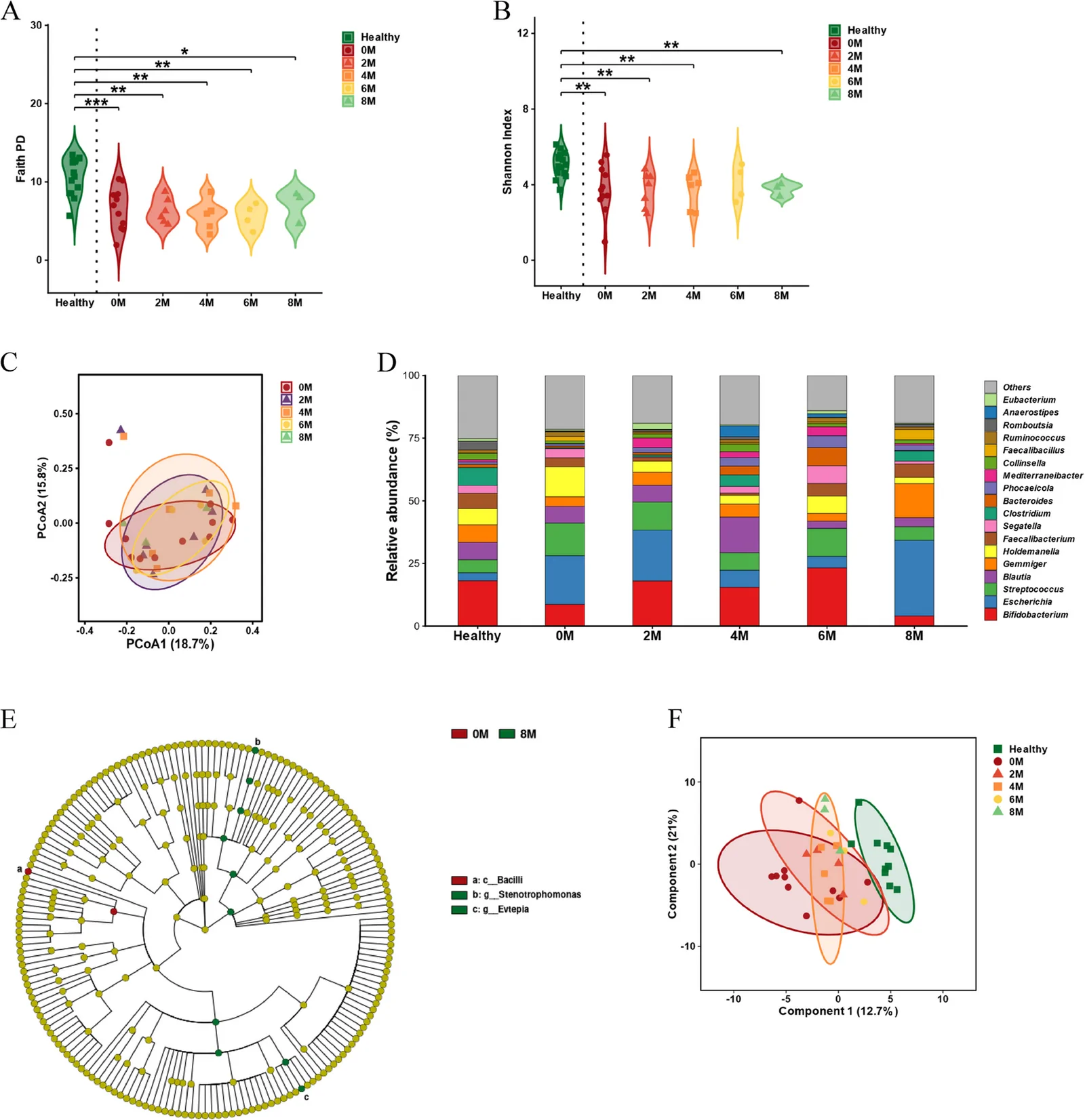
Fecal microbiota and metabolite study of longitudinally followed-up tuberculosis patients. Alpha diversity comparisons based on **A**) Faith PD, **B**) Shannon indices, and **C**) Principal coordinate analysis (PCoA) of unweighted Unifrac distance. **D**) Stacked bar plots representing the mean relative abundance (%) of the most abundant microbial taxa collapsed to the genus level. Taxa with mean abundance <1% and unclassified taxa were grouped as “Others’’.** E**) Cladogram representation of LEfSe analysis based on effect size (LDA score [log10] threshold of 2. Differences among study groups were assessed using the Kruskal-Wallis test (α = 0.05) and the Wilcoxon test (α = 0.05), with less stringent criteria for multi-class analysis. **F**) PLS-DA score plot for comparison of the global faecal metabolite profiles between 0,2,4,6,8 month and healthy subjects. Coloured circles represent 95% confidence intervals. Coloured dots represent individual samples

For the longitudinal study, a set of 10 ATB subjects were monitored for 8 months post initiation of the treatment, and samples were collected at 2, 4, 6, and 8 months. There were no significant changes in alpha and beta diversity over the course of treatment, whereas the significant difference decreased over time compared with healthy controls (Fig. 6A, B and C). Of particular interest to us were the reduced abundance of *Streptococcus* upon treatment and the increase in the abundance of opportunistic pathogens, *Escherichia* and *Stenotrophomonas* (Fig. 6D and E; Supplementary Figure S10). In follow-up cases, clustering in PLSDA revealed changes in the faecal metabolite profile at different time points after initiation of anti-tuberculosis treatment (Fig. 6F). A significant decrease in proline levels was observed at 8 months post-treatment, comparable to those of healthy controls (Supplementary Figure S11).

### Prediction of functional potential

**Fig. 7:**
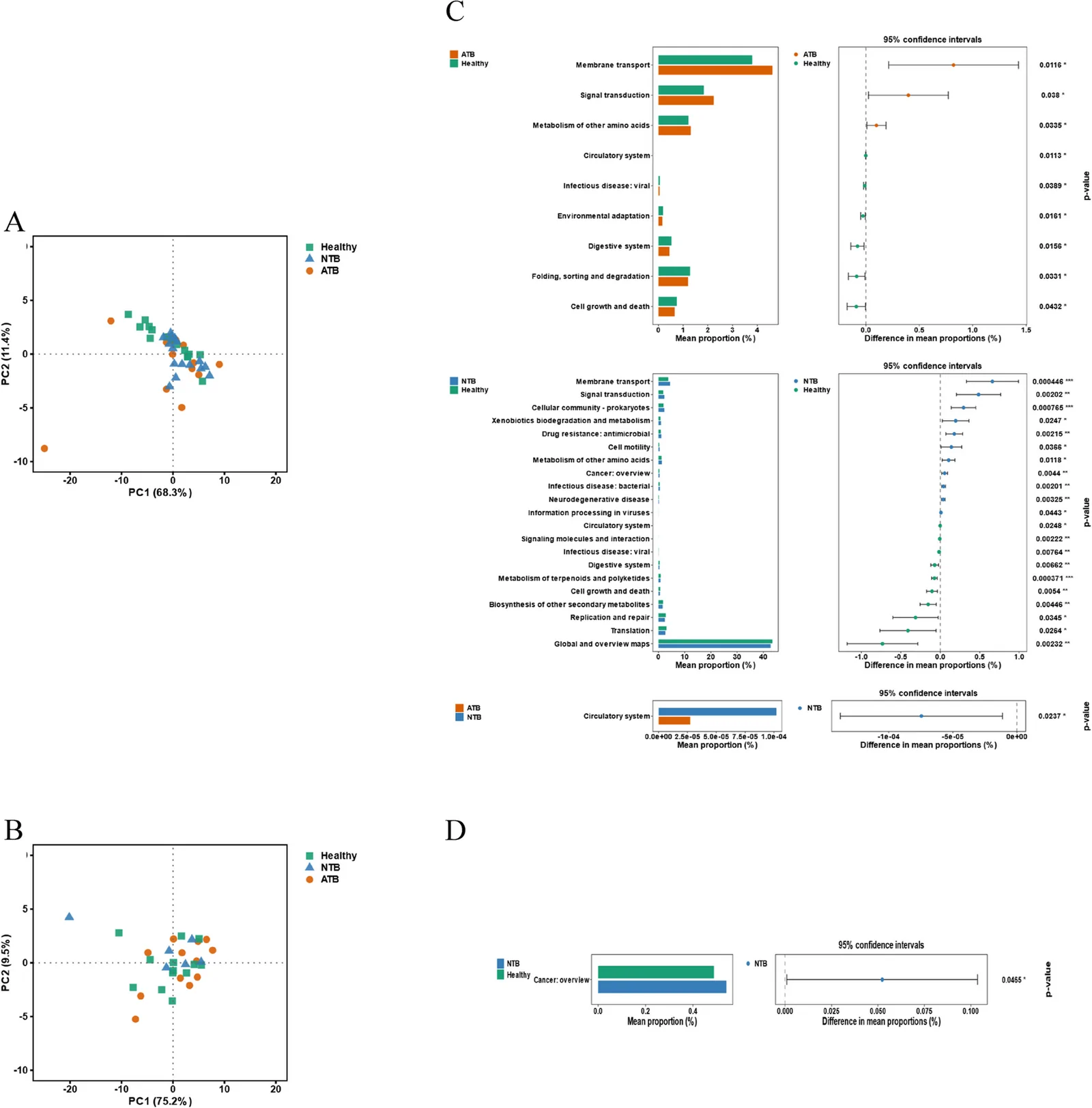
PICRUSt 2 analysis of study groups (active tuberculosis: ATB, healthy and non-tuberculosis: NTB). Statistical analyses were conducted in R (v 4.5.3). Principal Component Analysis (PCA) of KEGG Level 2 metabolic pathways showing functional differences among study groups from **A**) Site-I and **B**) Site-II. Differential abundance analysis of KEGG Level 2 pathways was performed using the Kruskal–Wallis H test followed by Dunn's post-hoc multiple-comparison test without multiple-testing correction. An extended error bar plot showed the mean proportions (%) of significantly different predicted functional categories at level 2 between the study groups **C**) site-I and** D**) site-II, using Welch’s t-test and the DP (Welch’s inverted test) for confidence intervals. Variance analysis revealed the abundance ratios of different functions across two sample groups. The point shows the difference between the proportions of functional abundance within the 95% confidence interval, and the value at the rightmost is the p-value. p<0.05 presents statistical significance

Variance analysis of KEGG metabolic pathways revealed differences in the functional genes in the microbiota of ATB and NTB from healthy NHAK groups (Fig. 7A). Of the 46 active KEGG pathways identified at level 2, 9 showed significant differences between ATB and healthy (*p* < 0.05), and 21 showed differences between NTB and healthy groups. Further, a significant difference was observed between ATB and NTB groups in circulatory system pathways. (Fig. 7C). In AGMC, study subjects showed similar clusters with a significant difference in the cancer overview metabolism pathway between NTB and healthy (Fig. 7B and D). Functional profiles were inferred using PICRUSt2 and represent predicted rather than experimentally validated metabolic capacities.

## Discussion

Most studies investigating gut microbiome dysbiosis in TB patients have focused on a single population. Multiple population studies are required to better understand the role of gut microbiota in TB pathogenesis and its association with disease outcomes. Therefore, the current study included participants from two distinct clinical sites to investigate the relationship between gut microbiota and TB across different patient populations. Gut dysbiosis is known to adversely affect host physiology and disrupt homeostatic processes. Previous studies have reported a significant reduction in gut microbial diversity and taxonomic numbers in patients with pulmonary TB compared to healthy controls [9, 22–24]. In this study, we characterized the microbial diversity, taxonomic composition, predicted metabolic function, and global fecal metabolome differences in the gut microbiota of drug-naive ATB subjects compared to those of NTB subjects (i.e., individuals with other disease conditions presenting similar clinical symptoms) and healthy controls. Furthermore, the study included a set of longitudinally followed-up ATB subjects who had completed their therapeutic interventions to evaluate microbiome and metabolome changes associated with the interventions.

The present study on gut microbiome analysis of ATB and NTB subjects revealed a lower alpha diversity than healthy controls, corroborating earlier reports [9, 22–25]. The ATB and NTB groups exhibited comparable microbial diversity; however, at site-I, the NTB group presented reduced diversity than healthy controls. Beta diversity analysis further demonstrated significant differences in the gut taxonomic composition, confirming intestinal microbiome changes in ATB patients. The richness in bacterial diversity is considered to have protective effects on metabolic and inflammatory diseases [26]. Therefore, the observed alterations in the intestinal microbiota of TB patients may indicate compromised anti-inflammatory potential and disrupted gut homeostasis.

At the genus level, a higher abundance of SCFAs-producing genera, including *Faecalibacterium* and *Bifidobacterium* was observed in the healthy controls [10, 24, 27]. SCFAs are essential for maintaining homeostasis, as they can induce either pro- or anti-inflammatory responses, depending on the signal transduction pathway [28]. *Bifidobacterium* has been associated with increased acetate concentration, while *Faecalibacterium* species is one of the most abundant butyrate-producing bacteria, and these metabolites protect the body from pathogens and inflammation [29]. Butyrate, for example, can activate G-protein-coupled receptors such as GPR43, GPR41, and GPR109a, leading to increased regulatory T cells (Tregs) and dendritic cell precursors, improved epithelial barrier function, and increased expression of anti-inflammatory cytokines such as IL-10. Phenylbutyrate (PBA), a butyrate derivative, induces the expression of antimicrobial peptides in lung epithelial cells and directly restricts the growth of *Mtb* in vitro and within macrophages [27, 30–33]. In the present study, the relative abundance of *Faecalibacterium* decreased in the ATB group compared to healthy, corroborating earlier reports [10]. Thus, this underpins the likely involvement of SCFAs in TB pathogenesis.

*Ligilactobacillus salivarius*, formerly named as *Lactobacillus salivarius* has been reported to have anti-inflammatory properties. It downregulates potent pro-inflammatory cascades (inhibiting TNF-α-induced IL-8 and IL-6 secretion) while preferentially expanding anti-inflammatory IL-10 tracking pathways, and thus could be a good probiotic candidate to reduce intestinal inflammation in IBD patients [34]. The marked depletion of *Ligilactobacillus* in our ATB patient cohort suggests a loss of regulatory commensals, potentially contributing to systemic and mucosal inflammation during tuberculosis infection.

Communication between the host and microbiome is partly mediated by metabolites, which profoundly influence the host physiology. Recent studies have uncovered that microbial metabolites are essential in regulating the immune system [35]. By altering the production of butyric acid and propionic acid, the intestinal microbiota leads to impaired immune function in TB patients [23]. Reports revealed that the intestinal microbial metabolite indole propionic acid (IPA) targets tryptophan to interfere with the metabolic activities of Mtb [36]. The fecal metabolome profile of ATB, healthy, and NTB subjects from both the study sites showed distinct group-specific clusters and a significant metabolic shift in ATB patients, corroborating earlier reports [23]. A set of 36 and 34 fecal metabolites showed significant alteration compared to healthy controls in ATB patients from Site I and Site II, respectively. ATB patients from NHAK showed a reduction in valeric acid, corroborating an earlier report [23]. Decreased valeric acid and increased malonic acid were also reported in ulcerative colitis, indicating the association of these molecules with intestinal inflammation [37]. Increased levels of N-acetyl putrescine in ATB suggest altered polyamine and amino acid metabolism in tuberculosis patients. Tyramine has been reported to benefit metabolic health by reducing insulin resistance in models of high-fat diet-induced obesity [38]. Oleic acid reduces intestinal inflammation in inflammatory bowel disease (IBD). Therefore, its decreased levels in ATB patients could directly indicate inflammation in tuberculosis patients [39]. Further, a set of 23 and 20 metabolites were significantly altered in ATB and NTB patients compared to healthy controls at site I and site II, respectively. A higher abundance of hydrocinnamic acid, N-acetyl putrescine, metronidazole, and a significantly lower KGDS/1, dihydrocinnamic acid, succinic acid, and valeric acid were observed in ATB and NTB subjects.

The study has a few limitations, including the lack of statistical sample size calculation and the heterogeneous composition of the NTB group, which may contribute to variability in microbiome and metabolome profiles. Furthermore, the longitudinal ATB cohort was relatively small. Future studies should include a substantially larger cohort, longitudinally followed beyond 6 months after completion of anti-TB therapy, to evaluate the long-term recovery and stability of the gut microbiome and metabolome.

Limited reports on the effects of anti-TB drugs on gut microbiota are available in the literature, and we monitored gut microbiota in a cohort of longitudinally followed-up drug-naïve TB patients. The decrease in abundance of *Streptococcus* indicate a partial recovery of the gut microbiota in treatment-completed TB patients. The emergence of opportunistic pathogen *Stenotrophomonas*,* Escherichia* and the gut commensal *Evtepia* indicates that the anti-TB treatment led to an increased abundance of microbes previously present at ultra-low baseline levels. Further, metabolomic analysis revealed partial restoration of proline levels following treatment. This recovery may be associated with an increased abundance of *Escherichia*, which utilizes proline for osmoregulation [40].

## Conclusion

The study findings demonstrate that the gut microbiome and metabolome show a partial shift towards baseline after completion of the anti-TB treatment regimen. The identified important microbes and metabolites may have translational potential and, after validation in a large population, could be used as probiotics or postbiotics to improve the prognosis of TB patients.

## Supplementary Information

Supplementary Material 1.

## Data Availability

The sequencing reads are deposited in SRA with BioProject Accession: PRJNA972267.

## Abbreviations

Mtb: Mycobacterium tuberculosis
TB: Tuberculosis
ATB: Active Tuberculosis
NTB: Non-tuberculosis
NHAK: Nagaland Hospital Authority, Kohima
AGMC: Agartala Government Medical College, Agartala
