# Supplementary material for: Gut microbiome dysbiosis and associated metabolome changes in tuberculosis patients resolve partially post-therapy
Source: BMC Microbiol. 2026 Nov 12;26:998. doi: 10.1186/s12866-026-05462-1 (PMC13756869; doi:10.1186/s12866-026-05462-1)
Supplement: Supplementary file 1 [file 12866_2026_5462_MOESM1_ESM.docx]

**Supplementary Information**

**Title:** Gut microbiome dysbiosis and associated metabolome changes in tuberculosis patients resolve partially post-therapy.

**Authors:**

Sukanya Sahu, Sandeep Rai Kaushik, Shweta Chaudhary, Amit Kumar Mohapatra, Rukuwe-u Kupa, Wetesho Kapfo, Sourav Saha, Ranjit Das, Nikhil Bhalla, Manjeet Kaur, Yuvaranjani Sivaji, Bhabatosh Das, Anjan Das, Vinotsole Khamo, Ranjan Kumar Nanda

**Supplementary Tables, Figures and Methods**

**Table S1.** Epidemiological details of all the study subjects used in the fecal metagenomics study. A/NTB: active/non-tuberculosis; M: months; NA: not available.

|  |  | Case-control study | | | | | | Follow-up study | | | | |
| --- | --- | --- | --- | --- | --- | --- | --- | --- | --- | --- | --- | --- |
|  |  | Site-I | | | Site-II | | | Site-I | | | | |
| Demographic details | Total | ATB | NTB | Healthy | ATB | NTB | Healthy | 0M | 2M | 4M | 6M | 8M |
| Population size | 107 | 14 | 16 | 15 | 12 | 6 | 13 | 10 | 7 | 6 | 4 | 4 |
| Mean age (range) in years | 43.2  (18-76) | 35.64  (20-60) | 40.56  (18-70) | 50.2  (25-68) | 45.5  (25-70) | 52.17  (42-61) | 46.7  (19-70) | 38.20  (20-76) | 45  (24-76) | 45  (27-76) | 32.25  (23-55) | 45  (24-56) |
| Gender (Female/Male) | 32/75 | 4/10 | 1/15 | 6/9 | 4/8 | -/6 | 3/10 | 4/6 | 5/2 | 3/3 | 2/2 | -/4 |
| Body mass index (kg/m^2^) |  | 21.93 | 23.94 | 25.24 | NA | NA | NA | 20.51 | NA | NA | NA | NA |
| Cough (y/n/na) | 60/18/29 | 14/-/- | 15/1/- | -/-/15 | 11/-/1 | 6/-/- | 1/4/8 | 10/-/- | -/5/2 | 3/2/1 | -/3/1 | -/3/1 |
| Expectoration (y/n/na) | 47/25/35 | 10/3/1 | 12/4/- | -/-/15 | 10/1/1 | 6/-/- | -/-/13 | 8/2/- | -/5/2 | 1/4/1 | -/3/1 | -/3/1 |
| Haemoptysis (y/n/na) | 20/58/29 | 5/9/- | 4/12/- | -/-/15 | 3/8/1 | 4/2/- | -/5/8 | 4/6/- | -/5/2 | -/5/1 | -/3/1 | -/3/1 |
| Chest pain (y/n/na) | 38/40/29 | 8/6/- | 9/7/- | -/-/15 | 10/1/1 | 2/4/- | -/5/8 | 9/1/- | -/5/2 | -/5/1 | -/3/1 | -/3/1 |
| Breathlessness (y/n/na) | 34/44/29 | 8/6/- | 9/7/- | -/-/15 | 8/3/1 | 2/4/- | -/5/8 | 7/3/- | -/5/2 | -/5/1 | -/3/1 | -/3/1 |
| Wheeze (y/n/na) | 28/50/29 | 8/6/- | 7/9/- | -/-/15 | 6/5/1 | -/6/- | -/5/8 | 7/3/- | -/5/2 | -/5/1 | -/3/1 | -/3/1 |
| Body temp.  (>37 °C/normal/na) | 34/45/28 | 8/6/- | 6/10/- | -/-/15 | 11/1/- | 4/2/- | -/5/8 | 5/5/- | -/5/2 | -/5/1 | -/3/1 | -/3/1 |
| Smoking (y/n/na) | 16/23/68 | -/4/10 | 3/13/- | -/-/15 | 8/3/1 | 3/3/- | 2/-/11 | -/-/10 | -/-/7 | -/-/6 | -/-/4 | -/-/4 |
| Alcoholism (y/n/na) | 20/37/50 | 1/3/10 | 7/9/- | 5/10/- | 5/6/1 | 2/4/- | -/5/8 | -/-/10 | -/-/7 | -/-/6 | -/-/4 | -/-/4 |
| Sputum AFB Test  (+ve/-ve/na) | 23/45/39 | 10/4/- | -/16/- | -/-/15 | 4/8/- | -/-/6 | -/-/13 | 9/1/- | -/5/2 | -/5/1 | -/3/1 | -/3/1 |
| GeneXpert test result  (+ve/-ve/na) | 35/24/48 | 12/1/1 | -/16/- | -/-/15 | 11/1/- | -/6/- | -/-/13 | 8/-/2 | 4/-/3 | -/-/6 | -/-/4 | -/-/4 |
| MGIT Culture test  (+ve/-ve/na) | 13/39/55 | 5/9/- | -/16/- | -/-/15 | 4/8/- | -/-/6 | -/-/13 | 4/4/2 | -/2/5 | -/-/6 | -/-/4 | -/-/4 |
| Rif resistance  (+ve/-ve/na) | 8/27/72 | -/12/2 | -/-/16 | -/-/15 | 8/3/1 | -/-/6 | -/-/13 | -/8/2 | -/4/3 | -/-/6 | -/-/4 | -/-/4 |

**Table S2.** Epidemiological details of study subjects used in fecal metabolomics. A/NTB: active/non-tuberculosis; M: months; NA: not available

|  |  | Case-control study | | | | | | Follow-up study | | | | |
| --- | --- | --- | --- | --- | --- | --- | --- | --- | --- | --- | --- | --- |
|  |  | Site-I | | | Site-II | | | Site-I | | | | |
| Demographic details | Total | ATB | NTB | Healthy | ATB | NTB | Healthy | 0 M | 2 M | 4 M | 6 M | 8 M |
| Population size | 94 | 13 | 12 | 15 | 11 | 6 | 13 | 9 | 4 | 4 | 3 | 4 |
| Mean age (range) in years | 45.2  (18-76) | 36.2  (20-60) | 40.2 (18-63) | 50.2  (25-68) | 47.1  (25-70) | 52.2 (42-61) | 46.7  (19-70) | 35.67  (20-76) | 52.8 (24-76) | 50.25 (27-76) | 35.3 (24-55) | 45  (24-56) |
| Gender (Female/Male) | 25/69 | 4/9 | 1/11 | 6/9 | 4/7 | -/6 | 3/10 | 3/6 | 2/2 | 1/3 | 1/2 | -/4 |
| Body mass index (kg/m^2^) |  | 22.33 | 24.35 | NA | NA | NA | NA | 20.27 | NA | NA | NA | NA |
| Cough (y/n/na) | 52/16/26 | 13/-/- | 11/1/- | -/-/15 | 10/-/1 | 6/-/- | 1/4/8 | 9/-/- | -/4/- | 2/2/- | -/2/1 | -/3/1 |
| Expectoration (y/n/na) | 41/21/32 | 10/2/1 | 8/4/- | -/-/15 | 9/1/1 | 6/-/- | -/-/13 | 7/2/- | -/4/- | 1/3/- | -/2/1 | -/3/1 |
| Haemoptysis (y/n/na) | 20/48/26 | 5/8/- | 4/8/- | -/-/15 | 3/7/1 | 4/2/- | -/5/8 | 4/5/- | -/4/- | -/4/- | -/2/1 | -/3/1 |
| Chest pain (y/n/na) | 33/35/26 | 7/6/- | 7/5/- | -/-/15 | 9/1/1 | 2/4/- | -/5/8 | 8/1/- | -/4/- | -/4/- | -/2/1 | -/3/1 |
| Breathlessness (y/n/na) | 30/38/26 | 8/5/- | 7/5/- | -/-/15 | 7/3/1 | 2/4/- | -/5/8 | 6/3/- | -/4/- | -/4/- | -/2/1 | -/3/1 |
| Wheeze (y/n/na) | 24/44/26 | 8/5/- | 6/6/- | -/-/15 | 5/5/1 | -/6/- | -/5/8 | 5/4/- | -/4/- | -/4/- | -/2/1 | -/3/1 |
| Body temp.  (>37 ºC/normal/na) | 31/38/25 | 8/5/- | 5/7/- | -/-/15 | 10/1/- | 4/2/- | -/5/8 | 4/5/- | -/4/- | -/4/- | -/2/1 | -/3/1 |
| Smoking (y/n/na) | 15/18/61 | -/3/10 | 3/9/- | -/-/15 | 7/3/1 | 3/3/- | 2/-/11 | -/-/9 | -/-/4 | -/-/4 | -/-/3 | -/-/4 |
| Alcoholism (y/n/na) | 12/24/58 | 1/2/10 | 5/7/- | -/-/15 | 4/6/1 | 2/4/- | -/5/8 | -/-/9 | -/-/4 | -/-/4 | -/-/3 | -/-/4 |
| Sputum AFB Test  (+ve/-ve/na) | 22/36/23 | 10/3/- | -/12/- | -/-/15 | 4/7/- | -/-/6 | -/-/13 | 8/1/- | -/4/- | -/4/- | -/2/1 | -/3/1 |
| GeneXpert test result  (+ve/-ve/na) | 33/20/41 | 11/1/1 | -/12/- | -/-/15 | 10/1/- | -/6/- | -/-/13 | 9/-/- | 3/-/1 | -/-/4 | -/-/3 | -/-/4 |
| MGIT Culture test  (+ve/-ve/na) | 13/32/49 | 5/8/- | -/12/- | -/-/15 | 3/8/- | -/-/6 | -/-/13 | 4/3/2 | -/2/2 | -/-/4 | -/-/3 | -/-/4 |
| Rif resistance  (+ve/-ve/na) | 8/23/63 | -/11/2 | -/-/12 | -/-/15 | 8/2/1 | -/-/6 | -/-/13 | -/8/1 | -/2/2 | -/-/4 | -/-/3 | -/-/4 |

**Table S3.** DADA2 summary statistics table for fecal samples of ATB, NTB, and healthy subjects from site-I

| **Subjects** | **Input** | **Filtered** | **Filtered (%)** | **Denoised** | **Merged** | **Merged (%)** | **Non-chimeric** | **Non-chimeric (%)** |
| --- | --- | --- | --- | --- | --- | --- | --- | --- |
| **id** |  |  |  |  |  |  |  |  |
| A11 | 156643 | 73976 | 47.23 | 73216 | 71122 | 45.4 | 60677 | 38.74 |
| A18 | 111758 | 40078 | 35.86 | 39097 | 36371 | 32.54 | 23593 | 21.11 |
| A2 | 120889 | 62615 | 51.8 | 61999 | 59468 | 49.19 | 51576 | 42.66 |
| A21 | 121746 | 64436 | 52.93 | 64354 | 64128 | 52.67 | 59582 | 48.94 |
| A22 | 110948 | 57129 | 51.49 | 54833 | 49214 | 44.36 | 38749 | 34.93 |
| A23 | 118916 | 62312 | 52.4 | 61578 | 58336 | 49.06 | 46299 | 38.93 |
| A24 | 132195 | 71915 | 54.4 | 71741 | 71295 | 53.93 | 70209 | 53.11 |
| A25 | 123711 | 63403 | 51.25 | 61617 | 56858 | 45.96 | 45560 | 36.83 |
| A27 | 136003 | 62861 | 46.22 | 60791 | 54801 | 40.29 | 40406 | 29.71 |
| A4 | 133774 | 65821 | 49.2 | 65263 | 62411 | 46.65 | 40863 | 30.55 |
| A5 | 124175 | 65745 | 52.95 | 63423 | 56703 | 45.66 | 44168 | 35.57 |
| A6 | 131797 | 71742 | 54.43 | 70175 | 64862 | 49.21 | 45666 | 34.65 |
| A8 | 126737 | 62504 | 49.32 | 62052 | 60601 | 47.82 | 51790 | 40.86 |
| H1 | 158247 | 69606 | 43.99 | 68343 | 63786 | 40.31 | 51278 | 32.4 |
| H10 | 140986 | 65637 | 46.56 | 63641 | 57827 | 41.02 | 44748 | 31.74 |
| H11 | 152763 | 82808 | 54.21 | 79842 | 71411 | 46.75 | 56405 | 36.92 |
| H12 | 135582 | 66591 | 49.11 | 63843 | 54894 | 40.49 | 38042 | 28.06 |
| H13 | 130412 | 60181 | 46.15 | 59500 | 56957 | 43.67 | 46667 | 35.78 |
| H14 | 144621 | 67549 | 46.71 | 65541 | 58817 | 40.67 | 46906 | 32.43 |
| H15 | 149448 | 69036 | 46.19 | 66645 | 60686 | 40.61 | 49997 | 33.45 |
| H2 | 153948 | 77894 | 50.6 | 75340 | 66917 | 43.47 | 48622 | 31.58 |
| H3 | 147012 | 74833 | 50.9 | 72567 | 65968 | 44.87 | 51943 | 35.33 |
| H4 | 114958 | 53565 | 46.6 | 52431 | 48335 | 42.05 | 38391 | 33.4 |
| H5 | 129370 | 65517 | 50.64 | 63841 | 58209 | 44.99 | 45414 | 35.1 |
| H6 | 146368 | 77202 | 52.75 | 73836 | 64795 | 44.27 | 49519 | 33.83 |
| H7 | 133658 | 72265 | 54.07 | 70252 | 61931 | 46.34 | 46819 | 35.03 |
| H8 | 130584 | 57585 | 44.1 | 55920 | 50871 | 38.96 | 37262 | 28.53 |
| H9 | 134410 | 64135 | 47.72 | 62029 | 54710 | 40.7 | 42466 | 31.59 |
| N1 | 119804 | 63565 | 53.06 | 62150 | 57596 | 48.08 | 45022 | 37.58 |
| N10 | 125944 | 74091 | 58.83 | 73995 | 73758 | 58.56 | 73133 | 58.07 |
| N11 | 120415 | 63885 | 53.05 | 62873 | 59166 | 49.14 | 48763 | 40.5 |
| N12 | 121383 | 63188 | 52.06 | 62379 | 59797 | 49.26 | 51481 | 42.41 |
| N13 | 115124 | 61054 | 53.03 | 58487 | 52823 | 45.88 | 43986 | 38.21 |
| N14 | 106069 | 46063 | 43.43 | 45422 | 43618 | 41.12 | 37652 | 35.5 |
| N15 | 131180 | 60743 | 46.31 | 60081 | 57705 | 43.99 | 46701 | 35.6 |
| N16 | 129139 | 58617 | 45.39 | 56215 | 49175 | 38.08 | 39180 | 30.34 |
| N2 | 126367 | 57896 | 45.82 | 56246 | 50836 | 40.23 | 39541 | 31.29 |
| N3 | 126675 | 66995 | 52.89 | 66720 | 65948 | 52.06 | 50764 | 40.07 |
| N4 | 116165 | 48867 | 42.07 | 47258 | 41713 | 35.91 | 34651 | 29.83 |
| N5 | 117123 | 53106 | 45.34 | 51942 | 48411 | 41.33 | 36325 | 31.01 |
| N6 | 109971 | 52557 | 47.79 | 51784 | 49180 | 44.72 | 38886 | 35.36 |
| N7 | 113941 | 59093 | 51.86 | 58056 | 53825 | 47.24 | 40684 | 35.71 |
| N8 | 145173 | 84340 | 58.1 | 83629 | 81277 | 55.99 | 68918 | 47.47 |
| N9 | 127391 | 62212 | 48.84 | 60934 | 56803 | 44.59 | 47384 | 37.2 |

**Table S4.** DADA2 summary statistics table of ATB, NTB and, healthy subjects from site-II

| **Sample-id** | **Input** | **Filtered** | **Filtered (%)** | **Denoised** | **Merged** | **Merged (%)** | **Non-chimeric** | **Non-chimeric (%)** |
| --- | --- | --- | --- | --- | --- | --- | --- | --- |
| A33 | 131153 | 60163 | 45.87 | 59409 | 56115 | 42.79 | 44110 | 33.63 |
| A34 | 114984 | 57129 | 49.68 | 56988 | 56820 | 49.42 | 55723 | 48.46 |
| A35 | 136555 | 74550 | 54.59 | 73798 | 70790 | 51.84 | 54166 | 39.67 |
| A36 | 135069 | 58885 | 43.6 | 58364 | 55511 | 41.1 | 45188 | 33.46 |
| A37 | 110909 | 66041 | 59.55 | 65168 | 62420 | 56.28 | 47111 | 42.48 |
| A38 | 130818 | 58054 | 44.38 | 57867 | 57332 | 43.83 | 55800 | 42.65 |
| A39 | 117128 | 54926 | 46.89 | 54251 | 51228 | 43.74 | 34659 | 29.59 |
| A40 | 112011 | 60649 | 54.15 | 60498 | 59900 | 53.48 | 54894 | 49.01 |
| A41 | 107967 | 58617 | 54.29 | 58414 | 57884 | 53.61 | 47396 | 43.9 |
| A42 | 115794 | 44010 | 38.01 | 43917 | 43639 | 37.69 | 42230 | 36.47 |
| A44 | 123830 | 69029 | 55.74 | 68280 | 65830 | 53.16 | 56355 | 45.51 |
| A45 | 118590 | 56970 | 48.04 | 56377 | 54750 | 46.17 | 48673 | 41.04 |
| H16 | 136091 | 65211 | 47.92 | 63936 | 59671 | 43.85 | 43409 | 31.9 |
| H17 | 160167 | 82642 | 51.6 | 80464 | 73915 | 46.15 | 57505 | 35.9 |
| H18 | 152826 | 84232 | 55.12 | 83868 | 75373 | 49.32 | 72764 | 47.61 |
| H19 | 137387 | 62309 | 45.35 | 60282 | 53488 | 38.93 | 33690 | 24.52 |
| H20 | 146790 | 74110 | 50.49 | 73356 | 71710 | 48.85 | 62636 | 42.67 |
| H21 | 140212 | 70048 | 49.96 | 68179 | 64108 | 45.72 | 52681 | 37.57 |
| H22 | 100598 | 42760 | 42.51 | 42403 | 41665 | 41.42 | 38227 | 38 |
| H23 | 141565 | 77973 | 55.08 | 76012 | 68296 | 48.24 | 50729 | 35.83 |
| H24 | 124155 | 67876 | 54.67 | 67769 | 63604 | 51.23 | 59008 | 47.53 |
| H25 | 138703 | 70311 | 50.69 | 68823 | 63812 | 46.01 | 48092 | 34.67 |
| H26 | 125942 | 62817 | 49.88 | 62302 | 60131 | 47.74 | 45533 | 36.15 |
| H27 | 113523 | 49914 | 43.97 | 49802 | 49547 | 43.64 | 46636 | 41.08 |
| H28 | 134344 | 58004 | 43.18 | 56346 | 51240 | 38.14 | 38826 | 28.9 |
| N17 | 130475 | 68817 | 52.74 | 67665 | 61479 | 47.12 | 50604 | 38.78 |
| N18 | 124349 | 65883 | 52.98 | 63502 | 57413 | 46.17 | 45807 | 36.84 |
| N19 | 113569 | 55417 | 48.8 | 54991 | 51458 | 45.31 | 44275 | 38.99 |
| N20 | 131080 | 62102 | 47.38 | 61402 | 58296 | 44.47 | 39665 | 30.26 |
| N21 | 145276 | 82340 | 56.68 | 82138 | 81053 | 55.79 | 69913 | 48.12 |
| N22 | 147903 | 67853 | 45.88 | 67701 | 66555 | 45 | 62566 | 42.3 |

**Table S5.** DADA2 summary statistics table of metagenomics data obtained from the longitudinally followed-up tuberculosis patients

| **Sample-id** | **Input** | **Filtered** | **Filtered (%)** | **Denoised** | **Merged** | **Merged (%)** | **Non-chimeric** | **Non-chimeric (%)** |
| --- | --- | --- | --- | --- | --- | --- | --- | --- |
| 02A12 | 135345 | 52268 | 38.62 | 50969 | 45322 | 33.49 | 35034 | 25.88 |
| 02A13 | 131136 | 58086 | 44.29 | 57292 | 53971 | 41.16 | 43347 | 33.05 |
| 02A14 | 127672 | 70080 | 54.89 | 68143 | 62729 | 49.13 | 48794 | 38.22 |
| 02A16 | 113279 | 58945 | 52.04 | 57787 | 52143 | 46.03 | 37958 | 33.51 |
| 02A26 | 158840 | 75425 | 47.48 | 75115 | 70901 | 44.64 | 61420 | 38.67 |
| 02A28 | 119065 | 63528 | 53.36 | 63042 | 61023 | 51.25 | 55880 | 46.93 |
| 02A9 | 116555 | 58025 | 49.78 | 56665 | 51426 | 44.12 | 37109 | 31.84 |
| 04A14 | 139950 | 82272 | 58.79 | 80751 | 75845 | 54.19 | 59939 | 42.83 |
| 04A16 | 127139 | 51656 | 40.63 | 50561 | 45762 | 35.99 | 32143 | 25.28 |
| 04A20 | 118348 | 54700 | 46.22 | 53926 | 51133 | 43.21 | 40997 | 34.64 |
| 04A26 | 114647 | 47702 | 41.61 | 47285 | 45858 | 40 | 37371 | 32.6 |
| 04A28 | 97656 | 50012 | 51.21 | 49482 | 47564 | 48.71 | 38994 | 39.93 |
| 04A3 | 123908 | 66180 | 53.41 | 65118 | 61821 | 49.89 | 46946 | 37.89 |
| 06A12 | 123856 | 61146 | 49.37 | 59494 | 53487 | 43.18 | 42982 | 34.7 |
| 06A13 | 127080 | 63396 | 49.89 | 62572 | 59540 | 46.85 | 48422 | 38.1 |
| 06A3 | 111343 | 54906 | 49.31 | 53943 | 50962 | 45.77 | 40173 | 36.08 |
| 06A30 | 121131 | 51831 | 42.79 | 51149 | 48719 | 40.22 | 36513 | 30.14 |
| 08A10 | 120608 | 66831 | 55.41 | 66087 | 63663 | 52.79 | 50916 | 42.22 |
| 08A13 | 130191 | 64927 | 49.87 | 63284 | 59132 | 45.42 | 48706 | 37.41 |
| 08A16 | 126473 | 62409 | 49.35 | 61076 | 58031 | 45.88 | 44978 | 35.56 |
| 12A13 | 127765 | 67837 | 53.1 | 67228 | 65457 | 51.23 | 53887 | 42.18 |
| A10 | 116816 | 69429 | 59.43 | 68885 | 66521 | 56.95 | 55319 | 47.36 |
| A12 | 129590 | 62183 | 47.98 | 59986 | 54220 | 41.84 | 40303 | 31.1 |
| A13 | 115359 | 49963 | 43.31 | 49370 | 47667 | 41.32 | 37200 | 32.25 |
| A14 | 123572 | 65118 | 52.7 | 62533 | 57378 | 46.43 | 45425 | 36.76 |
| A16 | 133105 | 72133 | 54.19 | 68986 | 61857 | 46.47 | 50810 | 38.17 |
| A17 | 119835 | 67344 | 56.2 | 67259 | 67178 | 56.06 | 67150 | 56.04 |
| A20 | 153189 | 82217 | 53.67 | 81006 | 76764 | 50.11 | 59698 | 38.97 |
| A26 | 121363 | 60036 | 49.47 | 59493 | 56349 | 46.43 | 42281 | 34.84 |
| A3 | 112020 | 50235 | 44.84 | 49371 | 46377 | 41.4 | 31332 | 27.97 |
| A7 | 123362 | 54306 | 44.02 | 52939 | 48625 | 39.42 | 38848 | 31.49 |
| A9 | 128263 | 58759 | 45.81 | 57011 | 52181 | 40.68 | 43245 | 33.72 |

**Table S6.** Important fecal metabolites (n=36) identified in drug naïve tuberculosis patients (ATB) from healthy controls recruited from site-I. FC: fold change

| **Name** | **FC** | **log_2_(FC: A/healthy)** | **Raw. pval** | **-log_10_(p)** |
| --- | --- | --- | --- | --- |
| Oleic acid | 0.024143 | -5.3722 | 8.86E-09 | 8.0524 |
| n-Pentadecanoic acid | 0.038869 | -4.6852 | 3.06E-07 | 6.5141 |
| Erythro-Pentonic acid | 152.19 | 7.2497 | 1.18E-06 | 5.927 |
| 1H-imidazol-2-amine | 127.93 | 6.9992 | 5.76E-06 | 5.2392 |
| 1H-Indole | 0.056675 | -4.1412 | 1.02E-05 | 4.9917 |
| N-acetylputrescine | 20.628 | 4.3665 | 1.92E-05 | 4.7159 |
| d-xylo-hexos-4-ulose | 0.010726 | -6.5427 | 2.06E-05 | 4.6855 |
| KGDS/1 | 0.033068 | -4.9184 | 2.81E-05 | 4.5509 |
| Cyclohexanecarboxylic acid | 0.022984 | -5.4432 | 2.88E-05 | 4.5412 |
| Hydrocinnamic acid | 36.24 | 5.1795 | 0.000126 | 3.8993 |
| 3-Hydroxyproline | 58.37 | 5.8672 | 0.000231 | 3.6355 |
| catecholpyruvate | 20.858 | 4.3826 | 0.001022 | 2.9904 |
| Metronidazole | 33.335 | 5.0589 | 0.001336 | 2.8741 |
| Methylmalonic acid | 8.2667 | 3.0473 | 0.001389 | 2.8572 |
| Malonic acid | 30.455 | 4.9286 | 0.001402 | 2.8533 |
| meso-Erythritol | 20.717 | 4.3727 | 0.001556 | 2.8081 |
| D-(+)-Xylose | 86.938 | 6.4419 | 0.001605 | 2.7945 |
| 3-Hydroxyphenylpropionic acid | 0.016194 | -5.9484 | 0.002724 | 2.5649 |
| L-Threitol | 37.693 | 5.2362 | 0.00307 | 2.5129 |
| Valeric acid | 0.28705 | -1.8006 | 0.003725 | 2.4289 |
| Tetracosanoic acid | 0.1017 | -3.2976 | 0.005138 | 2.2892 |
| Dipterin | 0.10425 | -3.2618 | 0.00554 | 2.2565 |
| Succinic acid | 0.14445 | -2.7914 | 0.005807 | 2.2361 |
| Propanoic acid | 10.369 | 3.3743 | 0.006344 | 2.1976 |
| 2-Piperidinone | 8.7779 | 3.1339 | 0.007475 | 2.1264 |
| d-Galactose | 5.5675 | 2.477 | 0.007526 | 2.1234 |
| Glutaric acid | 0.37526 | -1.414 | 0.007815 | 2.1071 |
| Tris-(2-methoxy-propyl)-amine | 6.954 | 2.7978 | 0.009037 | 2.044 |
| Dihydrocinnamic acid | 0.41769 | -1.2595 | 0.010053 | 1.9977 |
| D-Ribonic acid | 72.29 | 6.1757 | 0.010876 | 1.9635 |
| Tyramine | 0.16785 | -2.5747 | 0.013438 | 1.8717 |
| Proline | 21.883 | 4.4517 | 0.013719 | 1.8627 |
| L-Proline | 5.459 | 2.4486 | 0.028642 | 1.543 |
| Butyric acid, 2-amino- | 4.051 | 2.0183 | 0.030428 | 1.5167 |
| Cyclopentanecarboxylic acid, 1-amino- | 0.38096 | -1.3923 | 0.038463 | 1.415 |
| L-phenylalanine | 3.1201 | 1.6416 | 0.042951 | 1.367 |

**Table S7.** Important fecal metabolites (n=23) identified in non-tuberculosis (NTB) subjects with respect to healthy controls from site-I. FC: fold change

| **Name** | **FC** | **log_2_(FC:N/healthy)** | **raw.pval** | **-log_10_(p)** |
| --- | --- | --- | --- | --- |
| KGDS/1 | 0.024705 | -5.3391 | 1.36E-05 | 4.8653 |
| Hydrocinnamic acid | 101.51 | 6.6654 | 0.000116 | 3.9348 |
| Metronidazole | 24.953 | 4.6411 | 0.001011 | 2.9954 |
| Oleic acid | 0.16004 | -2.6435 | 0.001208 | 2.9178 |
| N-acetylputrescine | 29.619 | 4.8884 | 0.001279 | 2.8932 |
| Succinic acid | 0.10792 | -3.212 | 0.001646 | 2.7837 |
| 2-Bromosebacic acid | 0.1509 | -2.7283 | 0.001914 | 2.718 |
| 4-Ketoglucose | 6.4407 | 2.6872 | 0.002893 | 2.5386 |
| Methyl 6,9,12-hexadecatrienoate | 28.424 | 4.829 | 0.003246 | 2.4887 |
| n-Butylamine | 0.068748 | -3.8625 | 0.004219 | 2.3748 |
| 1H-imidazol-2-amine | 81.689 | 6.3521 | 0.004513 | 2.3455 |
| Cadaverine | 3.8195 | 1.9334 | 0.007818 | 2.1069 |
| N-(4-Bromo-2-fluoro-phenyl)-N'-(3-morpholin-4-yl-propyl)-oxalamide | 10.771 | 3.4291 | 0.011457 | 1.9409 |
| Dihydrocinnamic acid | 0.23936 | -2.0627 | 0.012606 | 1.8994 |
| D-Tryptophan | 2.5018 | 1.323 | 0.015016 | 1.8234 |
| D-Ribonic acid | 66.137 | 6.0474 | 0.015448 | 1.8111 |
| Valeric acid | 0.27572 | -1.8587 | 0.017787 | 1.7499 |
| Ala-Pro | 14.7 | 3.8778 | 0.018792 | 1.726 |
| Ã¡-Alanine | 3.9103 | 1.9673 | 0.028766 | 1.5411 |
| D-(+)-Xylose | 7.8628 | 2.975 | 0.034329 | 1.4643 |
| L-phenylalanine | 2.8153 | 1.4933 | 0.040021 | 1.3977 |
| Methylmalonic acid | 7.4325 | 2.8939 | 0.042666 | 1.3699 |
| Indole-3-carbinol | 2.9735 | 1.5721 | 0.047564 | 1.3227 |

**Table S8**.Important fecal metabolites (n=23) identified in drug naïve active tuberculosis patients (ATB) with respect to non-tuberculosis (NTB) controls from site-I. FC: fold change

| **Name** | **FC** | **log_2_(FC:A/N)** | **raw.pval** | **-log_10_(p)** |
| --- | --- | --- | --- | --- |
| n-Pentadecanoic acid | 0.038006 | -4.7176 | 1.05E-08 | 7.9774 |
| Erythro-Pentonic acid | 202.82 | 7.664 | 3.83E-06 | 5.4166 |
| 3-Hydroxyproline | 77.787 | 6.2815 | 0.000325 | 3.4876 |
| Tetracosanoic acid | 0.072661 | -3.7827 | 0.000629 | 3.2015 |
| 2-Bromosebacic acid | 7.614 | 2.9287 | 0.000831 | 3.0805 |
| Catecholpyruvate | 27.797 | 4.7969 | 0.00104 | 2.9828 |
| Cyclohexanecarboxylic acid | 0.017291 | -5.8538 | 0.001192 | 2.9239 |
| meso-Erythritol | 27.608 | 4.787 | 0.001383 | 2.8591 |
| Oleic acid | 0.15086 | -2.7288 | 0.004591 | 2.3381 |
| 2-Piperidinone | 11.698 | 3.5482 | 0.004626 | 2.3348 |
| Tris-(2-methoxy-propyl)-amine | 9.2672 | 3.2121 | 0.004715 | 2.3265 |
| 4-Ketoglucose | 0.15595 | -2.6808 | 0.005628 | 2.2496 |
| Methyl 6,9,12-hexadecatrienoate | 0.035337 | -4.8227 | 0.006334 | 2.1983 |
| d-xylo-hexos-4-ulose | 0.023967 | -5.3828 | 0.006415 | 2.1928 |
| Tyramine | 0.099271 | -3.3325 | 0.009317 | 2.0307 |
| 3-Hydroxyphenylpropionic acid | 0.049798 | -4.3278 | 0.01071 | 1.9702 |
| 1H-Indole | 0.064483 | -3.9549 | 0.011466 | 1.9406 |
| L-Threitol | 18.297 | 4.1935 | 0.011654 | 1.9335 |
| Dipterin | 0.10873 | -3.2012 | 0.012114 | 1.9167 |
| Malonic acid | 18.499 | 4.2094 | 0.014918 | 1.8263 |
| N-(4-Bromo-2-fluoro-phenyl)-N'-(3-morpholin-4-yl-propyl)-oxalamide | 0.093248 | -3.4228 | 0.019503 | 1.7099 |
| Ala-Pro | 0.068326 | -3.8714 | 0.030335 | 1.5181 |
| Talonic acid | 3.2213 | 1.6876 | 0.044174 | 1.3548 |

**Table S9.** Important fecal metabolites (n= 34) identified in drug naïve active tuberculosis patients (ATB) with respect to healthy controls from site-II study populations. FC: fold change

| **Name** | **FC** | **log_2_(FC:A/healthy)** | **raw.pval** | **-log_10_(p)** |
| --- | --- | --- | --- | --- |
| n-Propyl acetate | 36.649 | 5.1957 | 4.18E-07 | 6.3784 |
| 3-penten-2-one | 9.9257 | 3.3112 | 1.59E-06 | 5.7975 |
| Pyrazine | 13.342 | 3.7379 | 3.91E-06 | 5.4081 |
| Putreanine | 47.81 | 5.5792 | 1.05E-05 | 4.9785 |
| Tris(hydroxymethyl)aminomethane | 7.6626 | 2.9378 | 2.50E-05 | 4.6028 |
| proline | 84.412 | 6.3994 | 3.27E-05 | 4.4857 |
| 1-Amino-4-methylpiperazine | 22.628 | 4.5001 | 3.73E-05 | 4.4278 |
| Hydroxymalonic acid | 35.56 | 5.1522 | 6.89E-05 | 4.1617 |
| 1,2,3-Trimethoxycyclohexane | 10.161 | 3.3449 | 0.000461 | 3.336 |
| 2-Piperidinone | 31.266 | 4.9665 | 0.000501 | 3.3002 |
| Furethidine | 68.316 | 6.0942 | 0.000689 | 3.1616 |
| Acryl glycine | 19.735 | 4.3027 | 0.000698 | 3.1561 |
| D-Erythro-Pentopyranose | 269.49 | 8.0741 | 0.000999 | 3.0005 |
| Ribitol | 0.034304 | -4.8655 | 0.001734 | 2.7611 |
| N-ethoxycarbonyl-D-Alanine, | 23.509 | 4.5552 | 0.001793 | 2.7464 |
| D-Arabinonic acid | 7.4317 | 2.8937 | 0.001991 | 2.7009 |
| 2,3-2H-Quinolin-2-one | 6.2889 | 2.6528 | 0.002223 | 2.6532 |
| Citrulline | 26.864 | 4.7476 | 0.00226 | 2.646 |
| 4-Phenyl-1-[(2-trifluoromethyl-phenylcarbamoyl)-methyl]-piperidine-4-carboxylic acid ethyl ester | 13.002 | 3.7006 | 0.003242 | 2.4892 |
| Glutaric acid | 19.639 | 4.2957 | 0.003368 | 2.4727 |
| Tetracosanoic acid | 0.076318 | -3.7118 | 0.004743 | 2.3239 |
| Serine | 2.4295 | 1.2806 | 0.004903 | 2.3096 |
| L-Cysteine | 3.9236 | 1.9722 | 0.005353 | 2.2714 |
| Heptanoic acid | 0.026932 | -5.2145 | 0.005539 | 2.2566 |
| 4(equat)Ethyl-1-n-propyl-trans-decahydroquinol-4(axial)-ol | 4.2561 | 2.0895 | 0.005911 | 2.2283 |
| Ornithine | 6.4321 | 2.6853 | 0.006652 | 2.177 |
| Tetradecanoic acid | 0.39761 | -1.3306 | 0.007663 | 2.1156 |
| n-Butylamine | 6.5751 | 2.717 | 0.011045 | 1.9568 |
| Oxalic acid | 13.916 | 3.7986 | 0.011606 | 1.9353 |
| Pentanoic acid | 6.1351 | 2.6171 | 0.012043 | 1.9192 |
| Benzenepropanoic acid | 0.049651 | -4.332 | 0.012371 | 1.9076 |
| 4-Ketoglucose | 0.040562 | -4.6237 | 0.017581 | 1.755 |
| d-2-Aminobutyric acid | 5.7225 | 2.5166 | 0.022546 | 1.6469 |
| L-Tyrosine | 3.1435 | 1.6524 | 0.030762 | 1.512 |

**Table S10.** Important fecal metabolites (n=29) identified in non-tuberculosis subjects (NTB) with respect to healthy controls from site-II. FC: fold change

| **Name** | **FC** | **log_2_(FC:N/healthy)** | **raw. pval** | **-log_10_(p)** |
| --- | --- | --- | --- | --- |
| Citrulline | 55.641 | 5.7981 | 2.94E-06 | 5.5323 |
| Proline | 32.211 | 5.0095 | 0.000273 | 3.5633 |
| n-Propyl acetate | 26.897 | 4.7494 | 0.000364 | 3.4389 |
| Pyrazine | 8.7821 | 3.1346 | 0.000417 | 3.3799 |
| 3-penten-2-one | 20.924 | 4.3871 | 0.000496 | 3.3043 |
| Acryl glycine | 68.387 | 6.0956 | 0.000919 | 3.0365 |
| Acetic acid, 2-oxa-7-thia-tricyclo[4.3.1.0(3,8)]dec-10-yl ester | 3.0723 | 1.6193 | 0.003347 | 2.4753 |
| 1,2,3-Trimethoxycyclohexane | 9.2834 | 3.2147 | 0.003752 | 2.4257 |
| Thiazole, 2-hydroxy-4-methyl-5-(a-chloroethyl)- | 4.0579 | 2.0207 | 0.003866 | 2.4127 |
| L-Cysteine | 11.869 | 3.5691 | 0.004222 | 2.3745 |
| Ornithine | 21.261 | 4.4102 | 0.004408 | 2.3558 |
| Hypoxanthine | 5.8727 | 2.554 | 0.004597 | 2.3375 |
| N-ethoxycarbonyl-D-Alanine, | 6.9762 | 2.8024 | 0.006554 | 2.1835 |
| Propanetriol | 27.376 | 4.7748 | 0.008123 | 2.0903 |
| L-Valine | 4.7028 | 2.2335 | 0.008517 | 2.0697 |
| Tetracyclo[5.1.0.0(2,4).0(3,5)]octane, 8,8-dibromo-exo-6-[(trimethylsilyl)oxy]- | 11.347 | 3.5042 | 0.009144 | 2.0388 |
| Serine | 5.768 | 2.5281 | 0.009714 | 2.0126 |
| Pipecolic acid | 5.8191 | 2.5408 | 0.01066 | 1.9723 |
| l-Isoleucine | 4.1042 | 2.0371 | 0.018501 | 1.7328 |
| Ribitol | 0.034301 | -4.8656 | 0.018641 | 1.7295 |
| l-Aspartic acid | 4.0401 | 2.0144 | 0.021215 | 1.6734 |
| Benzilate | 0.16138 | -2.6315 | 0.026481 | 1.5771 |
| L-Tyrosine | 2.6499 | 1.406 | 0.028857 | 1.5398 |
| Tetracosanoic acid | 0.07631 | -3.712 | 0.034922 | 1.4569 |
| L-Leucine | 2.8217 | 1.4965 | 0.035041 | 1.4554 |
| L-Lysine | 2.1687 | 1.1168 | 0.036628 | 1.4362 |
| Heptanoic acid | 0.026929 | -5.2147 | 0.03845 | 1.4151 |
| DL-Phenylalanine | 2.6374 | 1.3991 | 0.039484 | 1.4036 |
| Glutamic acid | 2.2963 | 1.1993 | 0.043808 | 1.3584 |

**Table S11.** Important fecal metabolites (n=20) identified in drug naïve active tuberculosis patients (ATB) in comparison to non-tuberculosis (NTB) subjects from site II. FC: fold change

| **Name** | **FC** | **log2(FC:A/N)** | **raw.**  **pval** | **-log10(p)** |
| --- | --- | --- | --- | --- |
| Putreanine | 47.818 | 5.5795 | 0.001811 | 2.7421 |
| Tris(hydroxymethyl)aminomethane | 7.6639 | 2.9381 | 0.002972 | 2.527 |
| 1-Amino-4-methylpiperazine | 22.632 | 4.5003 | 0.003738 | 2.4273 |
| Hydroxymalonic acid | 35.566 | 5.1524 | 0.005284 | 2.277 |
| Acetic acid, 2-oxa-7-thia-tricyclo[4.3.1.0(3,8)]dec-10-yl ester | 0.32547 | -1.6194 | 0.007116 | 2.1478 |
| 4-Ketoglucose | 0.079184 | -3.6586 | 0.007204 | 2.1424 |
| Thiazole, 2-hydroxy-4-methyl-5-(a-chloroethyl)- | 0.24641 | -2.0208 | 0.008059 | 2.0937 |
| b-Aminoisobutyric acid | 2328.2 | 11.185 | 0.008784 | 2.0563 |
| Hypoxanthine | 0.17027 | -2.5541 | 0.009357 | 2.0289 |
| Propanetriol | 0.036526 | -4.7749 | 0.015283 | 1.8158 |
| 2-Piperidinone | 31.271 | 4.9668 | 0.015905 | 1.7985 |
| Tetracyclo[5.1.0.0(2,4).0(3,5)]octane, 8,8-dibromo-exo-6-[(trimethylsilyl)oxy]- | 0.088127 | -3.5043 | 0.016923 | 1.7715 |
| Furethidine | 68.328 | 6.0944 | 0.018942 | 1.7226 |
| D-Erythro-Pentopyranose | 269.54 | 8.0744 | 0.023185 | 1.6348 |
| Glutamic acid | 0.4358 | -1.1982 | 0.03259 | 1.4869 |
| D-Arabinonic acid | 7.433 | 2.8939 | 0.033672 | 1.4727 |
| 2,3-2H-Quinolin-2-one | 6.29 | 2.6531 | 0.035724 | 1.447 |
| Tetradecanoic acid | 0.4612 | -1.1165 | 0.043164 | 1.3649 |
| 4-Phenyl-1-[(2-trifluoromethyl-phenylcarbamoyl)-methyl]-piperidine-4-carboxylic acid ethyl ester | 13.004 | 3.7009 | 0.043765 | 1.3589 |
| Glutaric acid | 19.643 | 4.2959 | 0.04467 | 1.35 |

***Figure S1.*** *Age distribution between study groups (active tuberculosis: ATB, non-tuberculosis: NTB and healthy subjects in A) site-I and B) site-II. The age data expressed as mean ± standard deviation (SD) and the difference between groups was evaluated by two-tailed Student’s t test. **: p<0.01.*

B

A

A

B

**Figure S2.** Stacked bar plots showing the relative abundance of microbiota distribution in active tuberculosis patients (ATB), healthy and non-tuberculosis (NTB) subjects of A) site-I and B) site-II, with taxonomic features collapsed at the level of genus. Genus that remained unclassified or with lesser abundance are grouped as Others.

**Figure S3.** LEfSe analysis of active tuberculosis patients (ATB), non-tuberculosis (NTB) and healthy subjects of site-I based on effect size (LDA score [log 10] threshold of 2. Differences among study groups were obtained by the Kruskal-Wallis test (α = 0.05) and Wilcoxon test (α = 0.05) with less strict parameter for multi-class analysis. The mean and median relative abundance of the genus are indicated with solid and dashed lines, respectively.

**Figure S4.** LEfSe analysis of active tuberculosis patients (ATB), non-tuberculosis (NTB) and healthy subjects of site-I based on effect size (LDA score [log 10] threshold of 2. Differences among study groups were obtained by the Kruskal-Wallis test (α = 0.05) and Wilcoxon test (α = 0.05) with less strict parameter for multi-class analysis. The mean and median relative abundance of the genus are indicated with solid and dashed lines, respectively.

**Figure S5.** LEfSe analysis of active tuberculosis patients (ATB), non-tuberculosis (NTB) and healthy subjects of site-I based on effect size (LDA score [log 10] threshold of 2. Differences among study groups were obtained by the Kruskal-Wallis test (α = 0.05) and Wilcoxon test (α = 0.05) with less strict parameter for multi-class analysis. The mean and median relative abundance of the genus are indicated with solid and dashed lines, respectively.

**Figure S6.** Histogram showing the relative abundances of identified species in drug naïve tuberculosis patients (ATB), and controls (healthy and non-tuberculosis: NTB) of site-II by LEfSe analysis. The mean and median relative abundance of the genus are indicated with solid and dashed lines, respectively.

**Figure S7.** Histogram showing the relative abundances of identified species in drug naïve tuberculosis patients (ATB), and controls (healthy and non-tuberculosis: NTB) of site-II by LEfSe analysis. The mean and median relative abundance of the genus and species are indicated with solid and dashed lines, respectively.

F

E

B

A

**Figure S8.** Fecal metabolite analysis of study groups to identify important deregulated molecules from site-I. Volcano plots of A) ATB vs healthy (low/high: 15/21) C) NTB vs healthy (low/high: 7/16) E) ATB vs NTB (low/high: 13/10). Red dots indicate features that presented both a FC ≥ 2 and p-value ≤ 0.05. Important fecal metabolites (the top 25) qualifying VIP score >1 between B) ATB vs healthy D) NTB vs healthy and F) ATB vs NTB. Coloured boxes on the right indicate the relative concentrations of the corresponding metabolite in each study group.

D

C

F

D

B

E

C

A

**Figure S9.** Fecal metabolite analysis of active tuberculosis patients (ATB), non-tuberculosis (NTB) and healthy subjects of site-II. Volcano plots of A) ATB vs healthy (low/high: 6/28) C) NTB vs healthy (low/high: 4/25) E) ATB vs NTB (low/high: 8/12). Red dots indicate features that presented both a FC ≥ 2 and p-value ≤ 0.05. Rank of the different metabolites (the top 25) identified by the PLS-DA between B) ATB vs healthy D) NTB vs healthy F) ATB vs NTB, according to the VIP score on the x-axis. Coloured boxes on the right indicate the relative concentrations of the corresponding metabolite in each group.

**Figure S10.** LEfSe analysis of drug-treated followed-up tuberculosis patient samples revealed differential species abundance. Histogram of the relative abundances of genus and species in 0, 2, 4, 6, and 8 month subjects. The mean and median relative abundance of the species are indicated with solid and dashed lines, respectively.

**Figure S11.** Multivariate analysis of fecal metabolome profile of followed up tuberculosis patients at different months of completing treatment (0/2/4/6/8) and healthy samples using Metaboanalyst 5.0. Rank of the different metabolites (the top 25) identified by the PLS-DA. Coloured boxes on the right indicate the relative concentrations of the corresponding metabolite in each group.

**Supplementary Experimental Procedures**

**DNA extraction and 16S rRNA gene amplicon sequencing:** Fecal samples (200 mg) were taken in a pre-chilled microcentrifuge tube (MCT, 1.5 ml). The samples were vortexed after adding TE buffer (200 µl, 50 mM Tris-1 mM EDTA, pH 8.0) and glass beads (4 in total, 2.7 mm, Biospec). Lysozyme (50 µl, 10 mg/ml), mutanolysin (6 µl, 2 KU/ml), and lysostaphin (3 µl, 4 KU/ml) were added to the sample and incubated at 37˚ C for 1 hour (hr). To this reaction mixture, guanidinium thiocyanate (250 µl, 4M) was added, mixed for 45 sec and subsequently incubated at 37˚ C for 10 minutes (min) at 300 rpm in Thermomixer C (Eppendorf) after mixing with N-lauryl sarcosine (300 µl of 10 %).

After vortexing and a short spin of 16,000 g for a few seconds, samples were incubated at 70˚C for 1 hr. Then, zirconia beads (300 mg of 0.1 mm, Biospec) were added, followed by bead beating in two cycles of 30 seconds (sec) each, with a total program time of 2 min using a bead beater (Mini bead beater 16, Biospec Products Inc. USA). After bead beating, PVPP (15 mg) was added, vortexed, and then centrifuged at 16,000 g for 3 min. The supernatant was transferred to a fresh MCT, and the pellet was washed twice with 500 µl of Tris (50 mM)-EDTA (20 mM)-NaCl (100 mM)-PVPP (1%), and the resulting supernatants were pooled. To the supernatant, isopropanol (2 ml) was added, and the tubes were incubated at room temperature for 10 min after inverting for a few min. The reaction mixture was centrifuged at 16,000 g for 10 min to harvest the pellet, which was then dried at room temperature. To each sample, phosphate buffer: potassium acetate (9:1, 1 ml) was added and incubated overnight at -20°C. The thawed samples were centrifuged at 16,000 g for 30 min at 4˚C. The supernatant was transferred to another tube, and RNase A (4 µl, 10 mg/ml) was added before incubation at 37˚C for 30 min. Then sodium acetate (50 µl, 3M) was added to each tube, followed by ice-cold ethanol (1 ml, 96 %) and mixed by inverting the tubes a few times before incubating at room temperature for 5 min. These samples were then centrifuged at 16,000 g for 15 min at 4 ˚C, and after discarding the supernatant, the pellet was washed with ice-cold ethanol (70%). The dried pellet was resuspended in Tris (10 mM)-EDTA (1 mM) buffer (pH 8.0, 200 µl), and harvested genomic DNA was quantified using a Qubit 3.0 fluorometer (Invitrogen, USA).

Extracted genomic DNA was sent to Macrogen in South Korea for library preparation and sequencing. The libraries were prepared according to the Illumina 16S Metagenomic Sequencing Library protocols, after amplification of the V3-V4 region of the 16S rRNA gene. The input gDNA (2 ng) was Polymerase Chain Reaction (PCR) amplified with 5X reaction buffer, dNTP mix (1 mM), the universal forward and reverse PCR primer (500 nM each), and Herculase II fusion DNA polymerase (Agilent Technologies, Santa Clara, CA). The cycle condition for the 1^st^ PCR was 3 min at 95 °C for heat activation, followed by 25 cycles of 30 sec at 95 °C, 30 sec at 55 °C, and 30 sec at 72 °C, and a 5 min final extension at 72 °C. The universal primer pair with Illumina adapter overhang sequences used for the first amplification were as follows: V3-F: 5’-TCGTCGGCAGCGTCAGATGTGTATAAGAGACAGCCTACGGGNGGCWGCAG-3', V4-R: 5’- GTCTCGTGGGCTCGGAGATGTGTATAAGAGACAGGACTACHVGGGTAT CTAATCC-3. The 1^st^ PCR product was purified with AMPure beads (Agencourt Bioscience, Beverly, MA). Following purification, the PCR product (2 μl) was amplified for final library constructig using the index NexteraXT Indexed Primer. The cycle condition for the 2^nd^ PCR was the same as the 1^st^, except it was expanded for 10 cycles. The PCR products were purified with AMPure beads. The final purified products were then quantified using qPCR following the qPCR Quantification Protocol Guide (KAPA Library Quantification kits for Illumina Sequencing platforms) and qualified using the TapeStation D1000 ScreenTape (Agilent Technologies, Waldbronn, Germany).

**Derivatization of fecal metabolites:** The fecal samples (n=94) were randomized before processing, and data acquisition was carried out in multiple batches of 8-12 samples per batch. To protect carbonyl groups and reduce the number of tautomeric isomers, methoxyamine (20 μl) in pyridine (30 mg/ml) was added to the dried fecal metabolites, followed by vortexing for 30 sec and incubation at 37 °C with generous shaking (1000 rpm) for 90 min. The sample vials were inverted once to capture any solvent condensation at the cap surface, followed by a short briefly. To derivatize hydroxyl and amine groups of the extracted fecal metabolites to trimethylsilylated (TMS) forms, N-methyl-N-(trimethylsilyl) trifluoroacetamide (MSTFA; 80 μl) with trimethylchlorosilane (1%, TMCS) was added, followed by vortexing for 10 sec and incubation at 37 °C with shaking (1000 rpm) for 30 min. After derivatization, samples were centrifuged at 10,000 g for 5 min, and the supernatant was transferred to a 200 µl glass vial inserted in a 2 ml vial. The derivatized fecal metabolites were brought to room temperature, and mass spectrometry data acquisition was carried out within 24 h of derivatization. An equal volume of extracted fecal metabolites of all samples was pooled to prepare a quality control (QC) sample. Aliquots (300 µl each) of the QC were used for derivatization using the same procedure, and at least three QC samples were run in each batch.
